# Seamless phase 2/3 design for trials with multiple co-primary endpoints using Bayesian predictive power

**DOI:** 10.1186/s12874-024-02144-2

**Published:** 2024-01-17

**Authors:** Jiaying Yang, Guochun Li, Dongqing Yang, Juan Wu, Junqin Wang, Xingsu Gao, Pei Liu

**Affiliations:** 1 Department of Public Health, School of Medicine, Nanjing University of Chinese Medicine, 138 Xianlin Rd, Nanjing, 210023 China; 2 Department of Epidemiology and Biostatistics, School of Public Health, Southeast University, No.87 Dingjiaqiao, Nanjing, 210009 China

**Keywords:** Seamless phase 2/3 design, Co-primary endpoints, Bayesian predictive power, Conditional power

## Abstract

Seamless phase 2/3 design has become increasingly popular in clinical trials with a single endpoint. Trials that define success based on the achievement of all co-primary endpoints (CPEs) encounter the challenge of inflated type 2 error rates, often leading to an overly large sample size. To tackle this challenge, we introduced a seamless phase 2/3 design strategy that employs Bayesian predictive power (BPP) for futility monitoring and sample size re-estimation at interim analysis. The correlations among multiple CPEs are incorporated using a Dirichlet-multinomial distribution. An alternative approach based on conditional power (CP) was also discussed for comparison. A seamless phase 2/3 vaccine trial employing four binary endpoints under the non-inferior hypothesis serves as an example. Our results spotlight that, in scenarios with relatively small phase 2 sample sizes (e.g., 50 or 100 subjects), the BPP approach either outperforms or matches the CP approach in terms of overall power. Particularly, with *n*_1_ = 50 and *ρ* = 0, BPP showcases an overall power advantage over CP by as much as 8.54%. Furthermore, when the phase 2 stage enrolled more subjects (e.g., 150 or 200), especially with a phase 2 sample size of 200 and *ρ* = 0, the BPP approach evidences a peak difference of 5.76% in early stop probability over the CP approach, emphasizing its better efficiency in terminating futile trials. It’s noteworthy that both BPP and CP methodologies maintained type 1 error rates under 2.5%. In conclusion, the integration of the Dirichlet-Multinominal model with the BPP approach offers improvement in certain scenarios over the CP approach for seamless phase 2/3 trials with multiple CPEs.

**Supplementary Information:**

The online version contains supplementary material available at 10.1186/s12874-024-02144-2.

## Introduction

Recently, the efficiency and effectiveness of clinical trials have become increasingly crucial for both the pharmaceutical industry and public sectors. Adaptive design, which allows researchers to respond to interim data with adaptions such as futility stopping and sample size re-estimation, has been widely adopted in oncology drug development. According to Cerqueira et al. [1], the most prevalent type of adaptation is the seamless phase 2/3 design, accounting for 23.1% of all adaptive designs. In Bothwell’s research [2], this figure rose to 57%. Compared with traditional approaches that conducted phase 2 trials for learning and phase 3 trials to confirm treatment effect separately, the seamless phase 2/3 design combines these two stages into a single study with an interim analysis between them. Consequently, regulatory stand-by times between the two stages are skipped and sample sizes are saved. Several authors [3–7] have discussed the general concepts of seamless phase 2/3 design. While, seamless designs have been effectively implemented for years in clinical trials with a single primary endpoint, trials with multiple co-primary endpoints (CPEs) present more complexity and challenges, as the presence of additional endpoints elevates the likelihood of false negatives, thus larger sample sizes are required to guarantee sufficient power for each endpoint [8].

Using two or more endpoints as CPEs for efficacy evaluation is becoming increasingly common, particularly in the development biological products. Examples include the bivalent AC meningitis vaccine, tetravalent influenza vaccine, 9-valent HPV vaccine, 23-valent pneumococcal polysaccharide vaccine and so on. Trials with CPEs are defined as a success if and only if all endpoints meet the efficacy requirement simultaneously [9]. Although no adjustment is needed to control the Type 1 error rate [10, 11], the Type 2 error rate increases as the number of endpoints increases. As the sample size calculated to detect the effect on all of the endpoints is always larger than the sample size calculated for a single endpoint [8, 12], it becomes more appealing to incorporate features such as early stopping into seamless designs for trials with multiple CPEs compared to those with a single endpoint. By including stopping rules, researchers could save valuable resources and protect subjects from exposure to ineffective treatments by stopping the trial for futility if the experimental treatment appears to be ineffective. Consequently, this work focuses on seamless phase 2/3 designs for trials with CPEs. Futility assessment at interim analysis was included for early stopping.

A well-accepted approach for futility assessment at interim analysis is to use a conditional approach [13–17]. This conditional approach can further be divided into conditional power (CP) [13, 18], a frequentist approach, and Bayesian predictive power (BPP) [17, 19], a Bayesian approach. The former estimates the probability of rejecting the null hypothesis at the end of a clinical trial based on the information accumulated [13]. It requires an assumption of the true effect size derived from the observed or initial hypothesized values. Although easy to implement, the CP approach has been criticized [20, 21] for relying solely on success probabilities evaluated at a single value while ignoring the variability of treatment differences. One solution to this problem is the use of BPP [17, 19, 22–24], which averages the conditional power over a prior distribution of the true treatment effect, incorporating the uncertainty of the effect size using the Bayesian framework, as opposed to assuming a single fixed value as with conditional power. Choi et al. [22] and Spiegelhalter et al. [19] introduced the use of BPP for binary endpoints. Choi et al. [17] later extended this method to trials with continuous endpoints. Schmidli [25] proposed a seamless phase 2/3 design using BPP for trials with survival endpoints, and Kimani et al. [26] presented a dose-selection procedure for binary outcomes in seamless phase 2/3 trials where both efficacy and safety are considered. Despite extensive discussion, few studies have focused on the use of BPP in trials with multiple CPEs. In this paper, we aim to apply BPP approach to trials with CPEs.

Concerning CPEs, a clinical trial that has been published was used as an example. This trial evaluated the efficacy and safety of quadrivalent meningococcal tetanus toxoid-conjugate vaccine [27], wherein the seroresponses for meningococcal serogroups A, C, W, and Y were considered as CPEs. These endpoints cannot be regarded as independent endpoints that follow a binomial distribution, as they are typically positively correlated [28]. To solve the problem of multiple endpoints, Thall, Simon, and Estey [29] proposed a Dirichlet-multinomial model for monitoring both adverse events and efficacy outcomes in evaluating a single-arm clinical trial. This approach allows researchers to monitor multivariate discrete outcomes while considering the correlation among endpoints. Zhou, Lee and Yuan [30] further adapted this model to accommodate co-primary efficacy endpoints in a Bayesian optimal phase 2 design. In this paper, the Dirichlet-multinomial model is employed to accommodate the outcomes representing the combination of seroresponses results for four binary endpoints in a non-inferior seamless phase 2/3 trial.

## Methods

### Notation

**Fig. 1:**
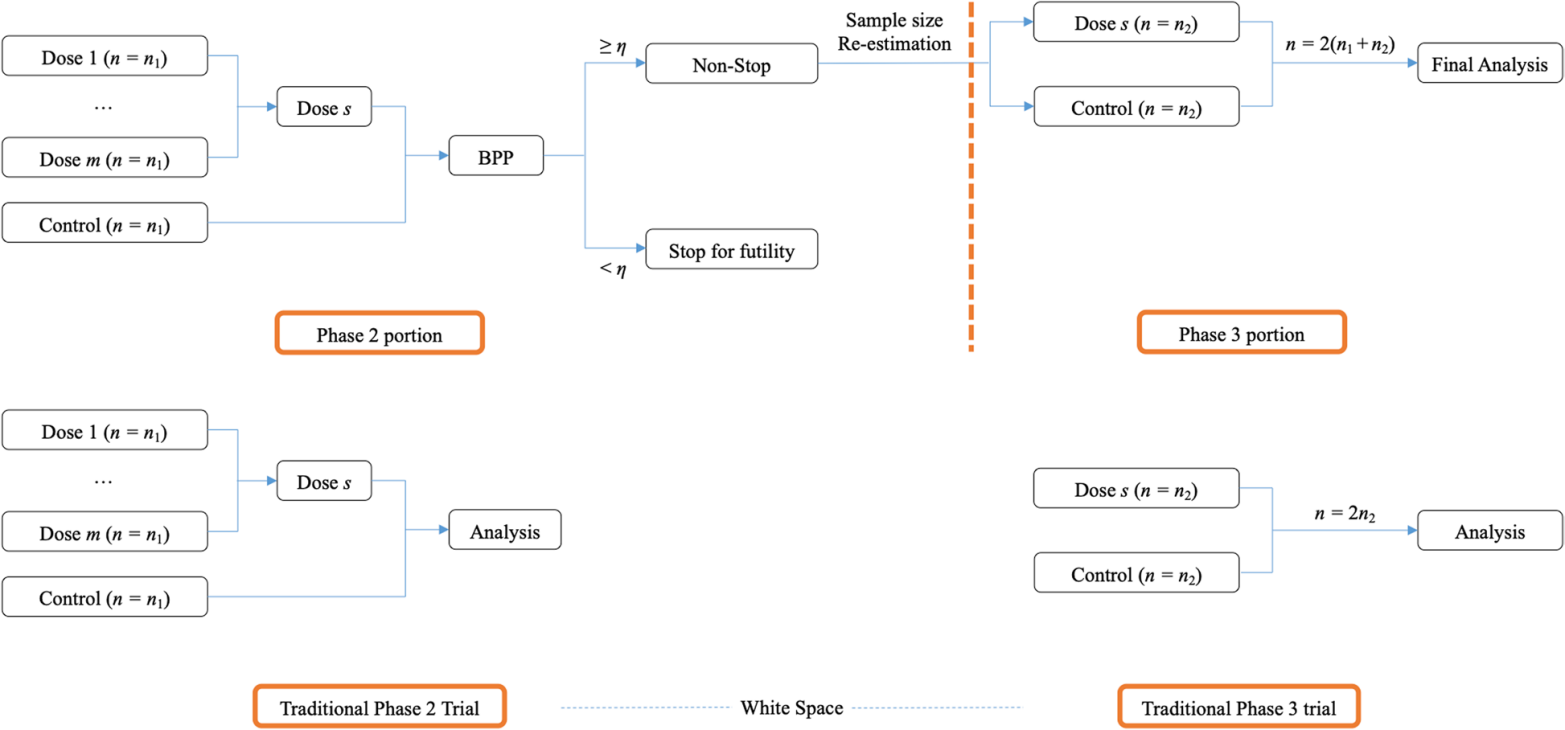
Study design schema of seamless phase 2/3 design and traditional design

Consider a seamless phase 2/3 vaccine trial conducted using *K* endpoints under the non-inferior hypothesis. This trial is defined as a success if and only if all the *K* endpoints meet the efficacy requirement simultaneously. Assume each group includes identical samples. Let *n*_1_ and *n*_2_ denote the sample sizes used for each group at the phase 2 and phase 3 stage, respectively. At the phase 2 stage, *M* doses of an experimental vaccine, *T*_*m*_ (*m* = 1,.., *M*), are evaluated against a positive control, *T*_*C*_. The most promising dose, *T*_*S*_, is selected for go/no-go decision-making with a pre-defined futility stop boundary, *η*. If the BPP for all dose groups based on the data from Phase 2 trials is less than *η*, it is concluded that all doses are in effective and should be stopped early. However, if the BPP for the dose group with the best efficacy is greater than or equal to *η*, that dose group will continue to Phase 3, and the required sample size for the Phase 3 stage is re-estimated. The final analysis is conducted based on the combined *p*-values, taking into account data from both phase 2 and phase 3 stages. If the combined *p*-value fall below the pre-specified significance level, the null hypothesis is rejected, indicating a successful trial; otherwise, the trial is considered a failure. This approach is denoted as BPP approach. As a comparative baseline, an alternative approach that utilizes CP for futility monitoring (denoted as CP method) will be implemented. A detailed design schema is presented in Fig. 1.

Assuming the same statistic, response rate, is used for interim monitoring and final analysis, ${\pi }_{mk}$ and ${\pi }_{Ck}$ represent the response rates for the experimental and control groups in terms of endpoint *k* (*k* = 1, …, *K*). For dose *m*, the vector of response rates ${{\varvec{\pi}}}_{m}$ = (${\pi }_{m1}$, …, ${\pi }_{mK}$) follows *K*-variate binomial distributions with the correlation matrix ***ρ***_*m*_,

$${\varvec{\rho}}m=\left[\begin{array}{ccc}1& \cdots & {\rho }_{m1K}\\ \vdots & {\rho }_{mkk{\prime}}& \vdots \\ {\rho }_{mK1}& \cdots & 1\end{array}\right]$$

where ${\rho }_{kk{\prime}}$ denotes the correlation coefficient between the *k*th and *k’*th endpoints for dose *m*.

To demonstrate non-inferiority of the trial at a one-sided significance level of *α* with a power of 1-*β*, the null hypothesis, *H*_0_: ${\pi }_{mk}-{\pi }_{Ck}\le \delta$ for at least one *k*, is tested against the alternatives *H*_1_: ${\pi }_{mk}-{\pi }_{Ck}> \delta$ for all *k*, where *δ* is the non-inferiority margin, *δ* $\in (-1, 0]$.

### Dirichlet-Multinomial model

The Dirichlet-multinomial model can be regarded as a generalization of the beta-binomial model when there are more than two categories. Suppose there is a trial with *n* subjects in each group. *K* binary endpoints are used as CPEs. A permutation based on the four endpoints produces *J* mutually exclusive outcomes that each subject may experience. The Dirichlet distribution is used as the prior distribution for the probabilities of the *J* outcomes. Let ${\varvec{\pi}}=\left({\pi }_{1},\dots ,{\pi }_{J}\right)$ represent the probability vector, and $\boldsymbol{\alpha }=\left({\alpha }_{1},\dots ,{\alpha }_{J}\right)$ denote the hyperparameters of the Dirichlet distribution. The prior distribution can be expressed as:

$${\varvec{\pi}}\sim Dir\left({\alpha }_{1},\dots ,{\alpha }_{J}\right)$$

$$p({\varvec{\pi}})\propto \prod_{j=1}^{J}{\pi }_{j}^{{\alpha }_{j}-1}$$

Given the observed data ${\varvec{x}}=\left({x}_{1},\dots ,{x}_{J}\right)$, where ${x}_{j}$ represents the number of subjects with a positive response for outcome *j*, the likelihood function of the Dirichlet-multinomial model can be written as:

$$L\left({\varvec{\pi}}|{\varvec{x}}\right)\propto \prod\limits_{j=1}^{J}{\pi }_{j}^{{x}_{j}}$$

Combining the prior distribution and the likelihood function, we obtain the posterior distribution, which is also a Dirichlet distribution. Let ${\boldsymbol{\alpha }}^{\mathrm{^{\prime}}}=\left({\alpha }_{1}^{\mathrm{^{\prime}}},\dots ,{\alpha }_{J}^{\mathrm{^{\prime}}}\right)$, where ${\alpha }_{j}^{\mathrm{^{\prime}}}={\alpha }_{j}+{x}_{j}$. The posterior distribution can be expressed as:

$${\varvec{\pi}}|{\varvec{x}} \sim Dir({\alpha }_{1}^{\mathrm{^{\prime}}}, \dots ,{\alpha }_{J}^{\mathrm{^{\prime}}})$$

$$p({\varvec{\pi}}|{\varvec{x}}) \propto \prod_{j=1}^{J}{\pi }_{j}^{{\alpha }_{j}^{\mathrm{^{\prime}}}-1}$$

The choice of hyperparameters ***a*** means the amount of information that is incorporated into the posterior model through the prior. Generally, $\left({\alpha }_{1},\dots ,{\alpha }_{J}\right)$ take the same value between zero and one [31].

In the presence of historical data, informative prior can be adopted with careful consideration and selection. Possible choices of informative priors and their impact on the results can be found in Supplementary Table 2 and 3 (see “Additional file 1” and “Supplement table”). In the absence of historical data, it is recommended to use non-informative priors. In this paper, Bayes-Laplace’s prior [32, 33], one of the most widely used non-informative priors, is taken with ${\alpha }_{1}$ = … = ${\alpha }_{J}$  = 1 so that the same density is given to each value of the probability vector ${\varvec{\pi}}$. Thus, the posterior distribution of ${\varvec{\pi}}$ is,

$${\varvec{\pi}}|{\varvec{x}} \sim Dir({1+x}_{1}, \dots ,1+{x}_{J})$$

### Test statistic for trials with multiple CPEs

At interim analysis, suppose an equal sample size, *n*_1_, is used for both the selected experimental dose (*S*) and the control group (*C*) in phase 2 stage. The *Z-*test statistic is employed to measure the effect size regarding the rate differences between group *S* and group *C* for a given endpoint *k* (*k* = 1, …, *K*), that is

$${Z}_{2k}=\frac{{\widehat{p}}_{Sk}-{\widehat{p}}_{Ck}-\delta }{{SE}_{{\widehat{\lambda }}_{k}}}$$

where ${\widehat{p}}_{Sk}$ and ${\widehat{p}}_{Ck}$ are the estimated response rates for the group *S* and group *C*, respectively. We have,

$${SE}_{{\widehat{\lambda }}_{k}}={\left\{{\widehat{p}}_{Sk}\left(1-{\widehat{p}}_{Sk}\right)/{n}_{1}+{\widehat{p}}_{Ck}\left(1-{\widehat{p}}_{Ck}\right)/{n}_{1}\right\}}^{1/2}$$

where ${SE}_{{\widehat{\lambda }}_{k}}$ is the standard error.

For trials with multiple CPEs, it is requisite to compute the *Z*-statistic for each of the *K* CPEs. Subsequently, the predictive probability is ascertained using the statistic associated with each endpoint. The endpoint with the smallest ${Z}_{2k}$ value, indicating the least effect size, is selected for sample size re-estimation.

### Type I error control and final analysis

Given the seamless Phase II/III design employed in this study, which falls under the category of confirmatory research, there's an imperative need to control the type I error at final analysis. Two primary sources contribute to the type I error in this context. Firstly, the selection of a dose from multiple doses during Phase II stage might not necessarily be the optimal one, leading to a potential type I error. Secondly, the interim analysis, which involves a peek into the data followed by possible sample size adjustments, also introduces an inherent risk of error.

To control the familywise type I error rate introduced by these processes, the Holm’s method [34] is employed initially to adjust the *p*-values derived from multiple comparisons during the phase II stage. This yields an adjusted *p*-value representing the phase II stage, thereby controlling the type I error from multiple dose comparisons. Subsequently, the inverse normal weighted combination test [35] is employed to combine this adjusted *p*-value with the one from the phase III stage, thereby ensuring control over the error introduced during interim analysis. Further details on these methodologies are presented in the appendix (see “Additional file 1”).

For endpoint *k*, the combined *p*-value at the final analysis is given by:

$${p}_{{\text{com}}{\text{k}} \, }=1-\Phi \left({w}_{1}\times {\Phi }^{-1}\left(1-{p}_{2k}^{\prime}\right)+{w}_{2}\times {\Phi }^{-1}\left(1-{p}_{3k}\right)\right)$$

where $\Phi$(⋅) represents the cumulative distribution function of the standard normal distribution, and ${\Phi }^{-1}$(⋅) denotes its quantile function. Here, ${p}_{2k}^{\prime}$ is the adjusted *p*-value for the endpoint *k* of the dose group selected from phase II stage, while ${p}_{3k}$ represents the *p*-value from phase III stage for the same endpoint. Given that only the dose group showcasing the best efficacy performance is chosen for phase III, ${p}_{2k}^{\prime}$ is derived from the dose with the smallest *p*-value among all treatment doses. According to the Holm’s method, ${p}_{2k}{\prime}$=*Mp*_2*k*_, where *p*_2*k*_ is the original *p*-value from phase 2, defined as *p*_2*k*_ = $1-\Phi ({Z}_{2k})$, *M* represents the number of treatment doses. Thus, under the null hypothesis, the type I error can be expressed as, $P\left({\bigcap }_{k=1}^{K}\left\{{p}_{comk}\le \alpha \right\}\right)$, where *α* is the one-sided significance level.

### Conditional power (CP)

In this section, only the core formulas are presented. For a comprehensive derivation and detailed explanation of the conditional power formula, see Wang, Keller, and Lan [18]. Their work extended Lan and Trost [36]’s approach to accommodate binary data in non-inferiority trials. This method computes the probability of trial success, conditional upon the observed data at an interim analysis and the assumption that future data will be consistent with current observations. Mathematically, for endpoint *k*​, given the interim analysis statistic ${Z}_{2k}$​ and a statistic for the final analysis *Z*_*k*_, the CP can be expressed as:

$$CP=P\left({Z}_{k}>c\mid {Z}_{2k}\right)$$

where *c* denotes a critical value associated with significance, while ${Z}_{2k}$ is the *Z* statistic derived from the phase II stage. In the context of this study, *c* = ${Z}_{1-\alpha }$.

Let *n*_*total*_ denote the total sample size of phase 2 and phase 3 stage for each group, such that *n*_*total*_ = *n*_1_ + *n*_2_. The fraction of accumulative information to the total information at the end of the phase 2 stage, symbolized by $\tau$, is:

1 $$\tau ={n}_{1}/{n}_{total}$$

Define the *B* value calculated at the information fraction $\tau$ as ${B}_{\tau }$. Assuming a linear relationship between statistics across stages, we have,

2 $${B}_{\tau }={Z}_{2k}\sqrt{\tau }$$

At final analysis, the information fraction $\tau'=\frac{n_{total}}{n_{total}}=1$, the statistic ${B}_{\tau {\prime}}=$ ${B}_{1}$. Consequently, the conditional power is formulated as:

$$CP=P\left({B}_{1k}\ge {Z}_{1-\alpha }\mid {B}_{\tau k}\right)$$

Here, ${B}_{1k}$​ and ${B}_{\tau k}$​ are the same statistics computed at different times, linearly related through their weighted sample size averages. Leveraging the properties of linear regression, we have, ${B}_{1}\sim N\left({B}_{\tau }/\tau ,1-\tau \right)$. Thus, with the data accumulated in the phase 2 stage, the CP of declaring non-inferiority at the end of the trial is,

3 $$\begin{array}{c}CP=P \left[{B}_{1}\ge {Z}_{1-\alpha }|{B}_{\tau }\right] \\ =P\left[N\left(\mathrm{0,1}\right)\ge \frac{{Z}_{1-\alpha }-\frac{{B}_{\tau }}{\tau }}{\sqrt{1-\tau }}\right]\\ =1-\Phi (\frac{{Z}_{1-\alpha }-\frac{{B}_{\tau }}{\tau }}{\sqrt{1-\tau }})\end{array}$$

where $\Phi (\cdot )$ denotes the standard normal distribution function.

Additionally, let *Z*_*CP*_ denote the test statistic for a desired CP, from (3), we have

4 $${Z}_{CP}=\frac{{Z}_{1-\alpha }-{B}_{\tau }/\tau }{\sqrt{1-\tau }}$$

With $\tau$ defined in Eq. (1) and ${B}_{\tau }$ defined in Eq. (2), Eq. (4) can be further expressed as

5 $${Z}_{CP}\sqrt{1-\frac{{n}_{1}}{{n}_{total}}}+{Z}_{\tau }\sqrt{\frac{{n}_{total}}{{n}_{1}}}-{Z}_{1-\alpha }=0$$

Let *n*_*min*_ represents the minimum sample size and *n*_*max*_ is the maximum sample size. The value of *n*_*total*_ could be obtained by solving Eq. (5). If the value obtained from Eq. (5) out of the range of [*n*_*min*_, *n*_*max*_], the corresponding minimum or maximum is selected as *n*_*total*_.

To evaluate the performance of CP approach, the following steps are executed in each iteration:

- Step 1. Binary correlation matrix construction and phase II data generation: This step corresponds to the initial procedures of the CP evaluation as detailed earlier.
- Step 2. Data generation for phase II stage: Draw n1 random samples from a K-variate binomial distribution using the aforementioned correlation matrix ρm.
- Step 3. Interim analysis: For each dose group m (m = 1, …, M) in the experimental arms, perform the following calculations: (a) For each endpoint, determine its Z-statistic and the corresponding p-value; (b) Compute the CP corresponding to each endpoint as per Eq. (3). Subsequently, adjust the p-values obtained for different doses m of the same endpoint k (k = 1, …, K) using the Holm's approach to account for multiple comparisons.
- Step 4. Decision-Making: For any given dose group m, identify the smallest CP value among the calculated CPs for the K endpoints as the CP value for that dose group, denoted as piCP (i = 1, …, M). Then, select the dose with the largest piCP. If the chosen piCP falls below a certain threshold, η, the trial will be deemed futile and be stopped. The decision will be recorded and the total sample size, ntotal, will be set to n1 from phase 2. Otherwise, the trial will proceed with steps 5–7.
- Step 5. Sample size re-estimation: If the trial continues, the sample size will be adjusted with an initial size nmin​. Iterative sample size adjustments and CP recalculations will be performed as per Eq. (5) until psCP meets a predefined target or the sample size reaches its upper limit nmax​.
- Step 6. Data generation for Phase III stage: Using the newly estimated sample size, new multivariate binary data is generated for the treatment and control groups.
- Step 7. Final analysis: Calculate the p-value for phase III stage. The trial’s success is ascertained based on the combined p-value. If the maximum combined p-value of all endpoints falls below α, H0 is rejected and the trial is deemed as a success; otherwise, it is deemed as a failure. Record the dose group chosen for the next phase, the exact sample size used, and the outcome of the final analysis concerning the rejection of H0​.

After *n*_*sims*_ iterations, tabulate the outcomes, including the frequency of each dose group identified as the optimal choice, the proportion of trials that progress to the next phase, the average *n*_*total*_, and the proportion in which *H*_0_​ is successfully rejected.

### Bayesian Predictive power (BPP)

BPP [19] is a measure used in Bayesian statistics to anticipate the probability of trial success, which combines the prior knowledge(captured by the prior distribution) with current observed data (represented by the likelihood function) to estimate the success probability of a trial. This estimation is achieved by computing the joint posterior predictive distribution over both future data and model parameters. In the context of our study, let *x*_0_ represent the data observed in the phase 2 stage and *X*_*f*_ represent the data that will be observed in the subsequent phase 3 trial. Let vector ***θ*** denote the unknown parameters which describe both the treatment effect of interest and any other nuisance parameters, e.g., ***θ* = **(*p*_*S*_, *p*_*C*_). BPP can then be conceptualized as the expectation of the success probability, computed over the joint posterior predictive distributions for future data *X*_*f*_ and parameters ***θ***. Mathematically, this is represented as:

$$BPP=\int p\left({X}_{f}\mid{\varvec{\theta}}\right)p\left({\varvec{\theta}}\mid {x}_{0}\right)d{\varvec{\theta}}$$

Here, $p\left({\varvec{\theta}}\mid {x}_{0}\right)$ denotes the posterior distribution of the parameter vector ***θ*** conditioned on the current data ${x}_{0}$. This distribution arises from the previously mentioned Dirichlet distribution. $p\left({X}_{f}\mid{\varvec{\theta}}\right)$ represents the likelihood of observing ${X}_{f}$ given a specific parameter vector ${\varvec{\theta}}$. While this likelihood can be perceived as the CP for a specific parameter vector ${\varvec{\theta}}$, it is essential to note that it’s not directly derived from the observed data. Rather, it is determined based on the posterior distribution of ${\varvec{\theta}}$, conditioned on the interim data ${x}_{0}$.

To evaluate the performance of the BPP approach, the following steps are executed in each iteration:

- Step 1. Binary correlation matrix construction and phase II data generation: This step corresponds to the initial procedures of the CP evaluation as detailed earlier.
- Step 2. Outcome computation: Based on the generated datasets, outcomes of all feasible binary combinations for both treatment and control groups are determined, yielding 2^*K*^ results per group. For instance, trials with four CPEs produce 16 mutually exclusive outcomes (see “Additional file 1” and “Supplement table”). These outcomes constitute the observed data ${\varvec{x}}=\left({x}_{1},\dots ,{x}_{{2}^{K}}\right)$ in the multinomial likelihood.
- Step 3. Posterior sampling for interim analysis: Given the observed data ${\varvec{x}}=\left({x}_{1},\dots ,{x}_{{2}^{K}}\right)$ and an assigned prior $\boldsymbol{\alpha }=\left({\alpha }_{1},\dots ,{\alpha }_{{2}^{K}}\right)$, 10,000 samples from the posterior for both treatment and control groups are drawn.
- Step 4. BPP computation: For each sample from the posterior distribution, the response rate will be estimated and its *Z*-statistic and the corresponding adjusted *p*-value will be determined. Then, the CP values corresponding to each endpoint will be computed using Eq. (3). These CP values are then averaged across all samples to determine the BPP for each endpoint within each dose group.
- Step 5. Decision-making: For any given dose group *m*, identify the smallest BPP value among the calculated BPPs for the *K* endpoints as the BPP value for that dose group, denoted as *p*_*i*_^*BPP*^ (*i* = 1, …, *M*). Then, select the dose with the largest *p*_*i*_^*BPP*^. If the chosen *p*_*i*_^*BPP*^ is below the threshold, *η*, the trial is considered a failure and terminated. This decision is documented analogously to step 4 in the CP approach. If not, the trial continues with steps 6–7.
- Step 6. Sample size re-estimation: The ensuing steps mirror the CP approach, with the exception that sample size re-estimation is conducted based on BPP, not CP.
- Step 7. Data generation for Phase III stage and final analysis: Utilizing the new sample size, datasets for the phase III stage are generated, followed by *p*-value calculation. The trial's success is ascertained via the combined *p*-value, and pertinent results are documented.

Upon completing *n*_*sims*_ iterations, relevant outcomes are documented.

In step 5, during the go/no-go decision, the stop boundary *η* is calibrated to be the maximum value within the range of 0 to 1, satisfying the following requirements: (1) The type 1 error rate is less than *α*; (2) The overall power is no less than 1—*β* when *p*_*S*_ = *p*_*C*_. The first rule aims to control the type 1 error rate when the null hypothesis is true, while the second rule is to control the type 2 error rate when the alternative hypothesis is true. The rationale behind selecting the highest cut-off value meeting these requirements is to terminate as many ineffective trials as possible in the interim analysis.

### Performance metrics

We calculated the following metrics for each scenario: (a) type 1 error rate; (b) overall power; (c) sample size; (d) stop percentage and (e) correct dose selection percentage. The type 1 error rate is defined as the percentage of “win” outcomes at the end of the entire study when the null hypothesis is true, where a “win” means rejecting the null hypothesis. A trial that doesn’t proceed to the phase 3 stage would be classified as a “fail”. Similarly, overall power is the percentage of “win” outcomes when the null hypothesis is false. The third metric, sample size, can be categorized into two types: the sample size used for each group throughout the entire trial (e.g., selected dose vaccine) and the sample size used for three experimental doses and one control group. Both types of sample size obtained from simulation are discussed in our article. Stopping probabilities, on the other hand, represent the futility stopping probability, refering to the percentage of simulated trials stopped due to futility at the end of the phase 2 stage. Last, the proportion of correct selection is defined as the percentage of simulated trials in which the best-performing dose is selected at the end of the phase 2 stage. To identify the best-performing dose, we compute the BPP (or CP) for each endpoint in each dose, taking the minimum value as the result of BPP (or CP) for the corresponding dose. The dose with the highest BPP (or CP) is selected as the best-performing dose.

## Results

To obtain the performance metrics, BPP was applied to a real vaccine trial in comparison with the CP approach. This study [37] investigates the non-inferiority of a quadrivalent meningococcal tetanus toxoid-conjugate vaccine with -10% as the inferiority margin (*δ* = -0.1). Seroresponse for meningococcal serogroups A, C, W, and Y were tested individually as CPEs, with *K* = 4. The definition of the Dirichlet-Multinomial model when there are 4 CPEs can be found in supplementary Table 1 (see “Additional file 1” and “Supplement table”). Suppose three doses of the experimental vaccine were used for dose selection, which represents low (*m* = 1), medium (*m* = 2) and high (*m* = 3), respectively. The seroresponse rates for the experimental group *m* and control group were defined as ***p***_*m*_ and ***p***_*C*_, respectively. In our study, the proportions of subjects achieving specific seroresponse for serogroups A, C, W, and Y at a pre-defined timepoint for control group is used as ***p***_*C*_, ***p***_*C*_ = (0.4246, 0.4965, 0.4478, 0.4339). Let ***p***_1_ = ***p***_3_ – 0.2, ***p***_2_ = ***p***_3_ – 0.1. To obtain type 1 error rates, we let ***p***_3_—***p***_*C*_ = -0.1. To obtain overall power, we let ***p***_3_—***p***_*C*_ > -0.1. The corresponding scenarios can be further divided into three types, (1) ***p***_3_ > ***p***_*C*_, (2) ***p***_3_ = ***p***_*C*_ and (3) ***p***_*C*_—0.1 < ***p***_3_ < ***p***_*C*_. For more details, see Table 1.

**Table 1:** Differences of seroresponse rates between high dose group and control group for the four co-primary endpoints

Scenarios			Endpoint 1	Endpoint 2	Endpoint 3	Endpoint 4
***Effective***	***p***_3_ > ***p***_*C*_	1	0.02	0.02	0.02	0.02
		2	0.02	0.02	0.02	0
		3	0.02	0.02	0	0
		4	0.02	0	0	0
	***p***_3_ = ***p***_*C*_	5	0	0	0	0
	***p***_*C*_—0.1 < ***p***_3_ < ***p***_*C*_	6	-0.02	0	0	0
		7	-0.02	-0.02	0	0
		8	-0.02	-0.02	-0.02	0
		9	-0.02	-0.02	-0.02	-0.02
***Ineffective***	***p***_3_—***p***_*C*_ = -0.1	-	-0.10	-0.10	-0.10	-0.10

Let the total sample size *n*_*total*_ = *n*_1_ + *n*_2_ = 450 for each group in the seamless 2/3 trial. The sample size for the phase 2 stage is set as *n*_1_ = (50, 100, 150, 200). At interim analysis, *n*_2_ is re-estimated based on BPP (CP) to achieve an overall power of 0.8. The minimum sample size for *n*_*total*_ is defined as *n*_min_ = 300 to obtain sufficient safety data. The maximum sample size is set to *n*_max_ = 1500. Equal correlation coefficient is assumed among endpoints as ${\rho }_{kk{\prime}}$ = (0, 0.3, 0.6) where *k* = 1,2,3,4, *k’* = 1,2,3,4, *k *$\ne$* k*’. Let the significance level be 0.025. The futility stop boundaries are calibrated under the scenario where ***p***_3_ = ***p***_*C*_, *n*_1_ = 100 and ${\rho }_{kk{\prime}}$ = 0, resulting in *η*_*BPP*_ = 0.01 and *η*_*CP*_ = 0.0018. The reason we chose different stopping boundaries for BPP and CP was that at *η* = 0.01, the overall power of the CP approach failed to meet the pre-specified requirement (2). Therefore, a smaller cut-off value was chosen for the CP approach to increase the possibility of moving to the next stage of a potentially effective trial. The performance of BPP and CP with different stop boundaries can be found in Supplementary Table 4.

### Type 1 error rate

Table 2 presents the simulated performance results for BPP in comparison with CP, taking into account common correlation coefficients among the four endpoints $\rho$ = (0, 0.3, 0.6) and the sample size allocated for each group at the phase 2 stage *n*_1_ = (50, 100, 150, 200), under the assumption that the null hypothesis is true. This result highlights that in each scenario, the type 1 error rates for both BPP and CP were less than 2.5%. Notably, when the correlation among endpoints is 0.6, the type 1 error rate is marginally higher compared to scenarios with correlations of 0 and 0.3. Taking the scenario where *n*_1_ = 50 as an example, the type 1 error rate for the CP approach is less than 0.01% (*ρ* = 0), 0.01% (*ρ* = 0.3) and 0.03% (*ρ* = 0.6), respectively.

**Table 2:** The performance of BPP in comparison with CP approach when the null hypothesis is true

*n*_1_	*ρ*	Type 1 Error (%)		Stop Percentage(%)		Sample size	
		**BPP**	**CP**	**BPP**	**CP**	**BPP**	**CP**
50	0	< 0.01	< 0.01	78.40	85.54	374.00	266.90
	0.3	< 0.01	< 0.01	68.22	75.65	526.46	413.34
	0.6	0.04	0.03	56.46	64.10	701.74	575.49
100	0	< 0.01	< 0.01	87.38	87.45	289.30	288.20
	0.3	< 0.01	< 0.01	78.17	78.32	427.35	423.77
	0.6	0.01	0.03	67.71	68.01	581.81	571.25
150	0	< 0.01	< 0.01	89.33	86.60	310.05	351.00
	0.3	< 0.01	< 0.01	80.30	77.08	445.50	493.46
	0.6	0.01	0.03	69.91	66.28	600.34	651.15
200	0	< 0.01	< 0.01	93.89	90.26	291.65	346.10
	0.3	< 0.01	< 0.01	85.93	81.64	410.93	475.06
	0.6	0.01	0.02	77.37	71.69	537.13	619.83

The percentage signs were omitted for type 1 error and stop percentage in the table; Sample size represents the sample size used for each group (eg. selected dose group) during the whole phase 2/3 trial

### Overall Power

**Fig. 2:**
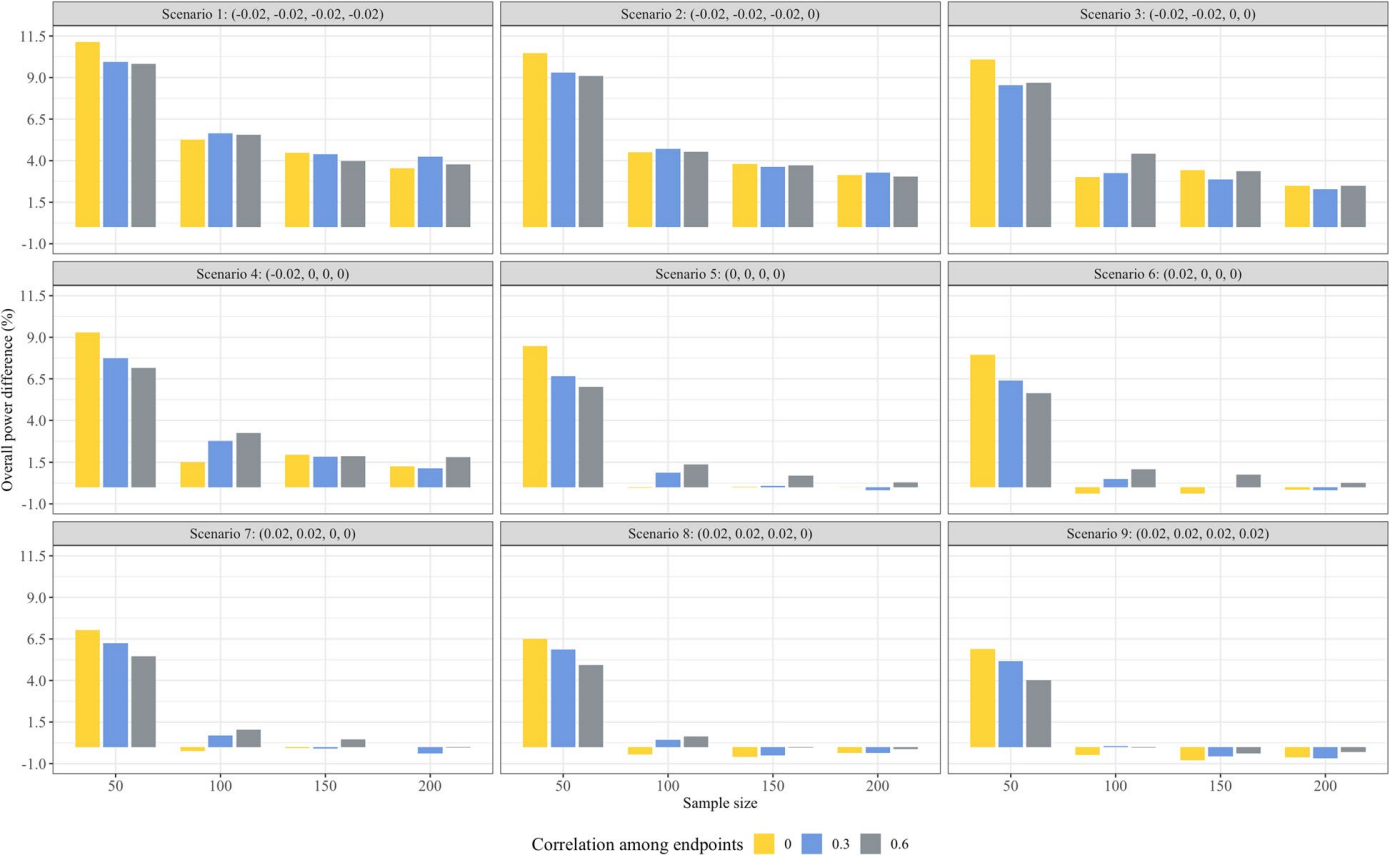
The Overall power difference between BPP and CP when the null hypothesis is false

**Fig. 3:**
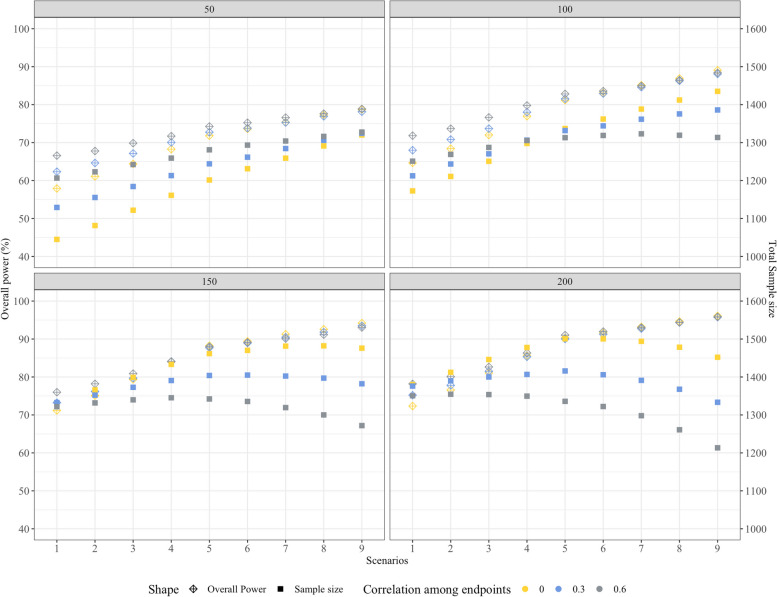
The behavior of overall power and total sample size of BPP when the null hypothesis is false

Figures 2 and 3 show the overall power performance of BPP in comparison with CP, considering common correlation coefficients among the four endpoints $\rho$ = (0, 0.3, 0.6) and sample size allocated for each group at the phase 2 stage *n*_1_ = (50, 100, 150, 200) across 9 different seroresponse scenarios when the null hypothesis is false. Figure 2 demonstrates the overall power difference between BPP and CP, while Fig. 3 depicts the behavior of overall power and total sample size for BPP. An intriguing observation from the data is that, in scenarios with *n*_1_ = 50, the overall power of BPP is 8.54% ($\rho$ = 0), 7.32% ($\rho$ = 0.3) and 6.76% ($\rho$ = 0.6) higher than the CP approach by an average across the nine seroresponse scenarios. This trend becomes more pronounced when ***p***_3_ is greater than or equal to ***p***_*C*_. For scenarios with *n*_1_ = 100, the power advantage of BPP over CP is 1.41% ($\rho$ = 0), 2.10% ($\rho$ = 0.3) and 2.43% ($\rho$ = 0.6). Meanwhile, when *n*_1_ = 150 or *n*_1_ = 200, the overall power of BPP consistently surpasses that of CP when ***p***_3_ is greater than or equal to ***p***_*C*_. Conversely, when ***p***_3_ is less than ***p***_*C*_, the power of BPP closely parallels CP, with differences lying within a narrow margin of ± 1%. Detailed performance of BPP and CP at interim analysis can be found in supplementary Table 5–8 (see “Supplementary table”).

### Sample size

Table 2 presents the sample size performance of both BPP and CP approaches when the null hypothesis is true. Table 3 displays the average sample size differences between the BPP and CP approaches across various seroresponse scenarios with $\rho$ = (0, 0.3, 0.6) and *n*_1_ = (50, 100, 150, 200) when the null hypothesis is false. From Table 2, we can see that as the stopping percentage increases, the sample size required for the study decreases. Under the null hypothesis, for scenarios with *n*_1_ = 50, BPP generally required more subjects compared to CP. Notably, in the case where $\rho$ = 0.6, the difference in sample size is 126.25. For scenarios with *n*_1_ = 100, the sample size necessitated by BPP are comparable to those by CP, with a marginal difference not exceeding 11 subjects. However, in scenarios where *n*_1_ = 150 or *n*_1_ = 200, BPP enrolls fewer subjects. Specifically, in scenario where *n*_1_ = 200 and $\rho$ = 0.6, BPP saves up to 82.70 subjects compared to CP.

**Table 3:** The performance of BPP in comparison with CP when the null hypothesis is false

Items	*ρ*	Mean^#^			
		***n***_**1**_** = 50**	***n***_**1**_** = 100**	***n***_**1**_** = 150**	***n***_**1**_** = 200**
Stop percentage difference^*^ (%)	0	-8.07	1.58	2.53	3.20
	0.3	-6.29	1.27	2.22	2.89
	0.6	-4.92	0.96	1.83	2.24
	total	-6.43	1.27	2.19	2.78
Sample size difference^*+^	0	143.97	23.85	31.86	39.74
	0.3	155.63	63.61	64.55	61.12
	0.6	184.22	102.65	93.04	83.75
	total	161.28	63.37	63.15	61.54
Correct dose selection (%)	0	90.94	96.70	98.71	99.54
	0.3	89.19	95.37	98.03	99.26
	0.6	87.48	94.42	97.20	98.86
	total	89.20	95.50	97.98	99.22

^*^represents the difference of BPP and CP, Value = BPP – CP; + : Sample size difference represents the difference of sample size used for each group (eg. selected dose group) during the whole phase 2/3 trial. ^#^: represents the average value of the nine different seroresponse scenarios

Referring to Table 3, it is generally observed that the sample sizes required by BPP surpass those of CP. Moreover, as the correlation coefficient among endpoints increases, the sample size needed for BPP amplifies, widening the gap from CP. Supplementary Table 5–8 (see “Supplementary table”) provide detailed information on the sample size used for each group across various scenarios when the selected experimental dose is non-inferior to the control vaccine. Additionally, Fig. 3 displays the overall power behavior of the BPP approach, along with the associated total sample sizes for different scenarios when the null hypothesis is false. Particularly, when *n*_1_ = 50, as the differences between the experimental and control groups decrease, the BPP method's required sample size incrementally grows. The trend intensifies with scenarios higher correlations. With *n*_1_ = 100, as the differences narrow, the rate of increase in sample size for BPP slows down, and a pronounced decrement is evident with higher correlations. For *n*_1_ = 150 or *n*_1_ = 200, the BPP method's sample size initially shows a mild rise followed by a decline as the difference between the experimental and control group narrows. Significantly, at *n*_1_ = 150 or *n*_1_ = 200, when ***p***_3_ is less than ***p***_*C*_, for a given scenario, the larger the correlation among endpoints, the lesser the sample size required.

### Stop Percentage

Table 2 presents the futility stop percentages of BPP and CP across correlation coefficients, $\rho$ = (0, 0.3, 0.6), and sample size at phase 2 stage, *n*_1_ = (50, 100, 150, 200), when the null hypothesis is true. From the table, we can see that the probability of BPP correctly stopping ineffective trials at interim analysis increases as the sample size used at the phase 2 stage increases. In contrasting different correlation scenarios for both BPP and CP approaches, the probability of correctly stopping an invalid trial at the end of phase 2 is highest when the endpoints are independent of each other. Supplementary Table 5–8 (see “Supplementary table”) detail the futility stop percentages of BPP and CP under each scenario when the null hypothesis is false, revealing similar results. For *n*_1_ = 50, under any scenarios, BPP's likelihood of erroneously terminating trials early surpasses CP's by a minimum of 3.69%. As *n*_1_ increases to 100 or more, the early stoppage probability for BPP slightly ecceeds that of CP. Specifically, under the scenario where *ρ* = 0 and the trial and control groups are most aligned, the difference in early stop percentage between BPP and CP peaks, reaching 1.74% (*n*_1_ = 100), 4.14% (*n*_1_ = 150), and 5.76% (*n*_1_ = 200). Table 3 provides the average stop percentage difference between the BPP and CP approaches for $\rho$ = (0, 0.3, 0.6) and *n*_1_ = (50, 100, 150, 200) in 9 scenarios when the null hypothesis is false. In general, the stop percentage difference between the BPP and CP approaches is -6.43%% (*n*_1_ = 50), 1.27% (*n*_1_ = 100), 2.19% (*n*_1_ = 150) and 2.78% (*n*_1_ = 200).

### Correct dose selection

Table 3 reveals that the probability of correct dose selection increases with a larger sample size used at the phase 2 stage and a smaller correlation among endpoints. In general, the average percentage of correct dose selection across different correlation and seroresponse scenarios are 89.20% (*n*_1_ = 50), 95.50% (*n*_1_ = 100), 97.98% (*n*_1_ = 150) and 99.22% (*n*_1_ = 200).

## Discussion

In this paper, we proposed a BPP approach to redesign a previously published quadrivalent meningococcal tetanus toxoid-conjugate vaccine trial, assuming it to be a non-inferior seamless phase 2/3 vaccine trial with four CPEs. The final analysis was conducted using a Bayesian approach. The performance of the BPP was evaluated for correlations among endpoints of 0, 0.3, and 0.6, and for phase 2 sample sizes of 50, 100, 150, and 200, in scenarios where the null hypothesis was true and false, using the CP approach for comparison purpose. The results presented in this paper indicate that when the sample size used for the phase 2 stage is relatively small (e.g., *n*_1_ = 50 or 100), the BPP approach either outperforms or matches the CP approach in terms of overall power when the experimental vaccine is non-inferior to the control vaccine. However, it requires larger sample sizes than the CP approach due to a lower early stop percentage if the experimental vaccine is inferior to the control vaccine. On the other hand, when more subjects are enrolled in the phase 2 stage (e.g., *n*_1_ = 150 or 200), the BPP method exhibits a higher probability of correctly stopping a futile trial compared to the CP method, all the while maintaining superior or equivalent overall power performance.

The most notable difference between the two approaches lies in their respective methodologies for estimating trial success. The BPP approach determines the probability of trial success based on the overall distribution of the seroresponse rates of the experimental and control group across each endpoint, considering the variability of treatment differences for a more comprehensive assessment. In contrast, the CP approach evaluates each endpoint separately based on its interim (*Z*_2*k*_) and final analysis statistics (*Z*_*k*_), without integrating the correlations among endpoints. This singular approach, anchored in the foundational principles of Wang, Keller, and Lan [18], might overlook the intricate interplay among endpoints. Although a binary correlation matrix is created during data generation, it remains external to CP's computational purview. Subsequent findings underscore that the Dirichlet-Multinomial model’s application within the BPP framework not only improves overall power performance but also reduces the likelihood of prematurely stopping trials, particularly in positively correlated endpoint scenarios. Dirichlet conjugate distributions also reduce the computational burden of the BPP approach, enhancing its applicability for clinical trials. Additionally, seamless phase 2/3 trials with multiple CPEs could shorten the overall trial duration, as is the case for trials with a single endpoint.

As demonstrated in this paper, the stop percentage of the BPP exhibits high sensitivity to the correlation among endpoints. Figure S1 depicts the density curve of the BPP and CP (see “Supplementary figure”). From this figure, it is evident that when endpoints are independent of each other, the BPP values tend to cluster at a lower level, leading to higher probabilities of early stop. In contrast, scenarios with a correlation of 0.6 among endpoints show a more dispersed BPP distribution, thereby reducing the likelihood of mistakenly stopping early for futility. This phenomenon can be attributed to the fact that when endpoints exhibit a high correlation (e.g., 0.6), the seroresponse rates for the four correlated endpoints generated by a random process become relatively aligned, making extreme outcomes less probable. After 10,000 iterations, we derived 10,000 sets of consistent seroresponse rates. Consequently, the distribution of the trial success probability computed at each iteration appears more uniform when there’s a high correlation among endpoints. As the probability of early stopping decreases, the overall power increases, and the total sample size decreases. Furthermore, the distribution of the BPP values when correlation is high, alongside the CP values derived from 10,000 simulations, both exhibit a bimodal pattern with peaks at both extremities. Such a U-shaped distribution aligns with plausible scenarios observed in clinical development [38]. Nonetheless, this distribution profile tends to induce a higher early stop for futility probability compared to a unimodal distribution, underscoring the need for further exploration and thoughtful integration in trial designs.

When selecting a sample size for the phase 2 stage, it’s essential to conduct a meticulous assessment. If the sample size used in the phase 2 stage is inadequate, there’s a risk that the optimal dose might not be identified for the subsequent phase 3 stage. In our simulation scenarios, although utilizing a smaller initial sample size can result in some sample savings — specifically, the BPP method can save an average of 155.45 subjects per group when *n*_1_ = 50 compared to *n*_1_ = 200 — we caution against relying on too small a sample size in the early phase. This is due to the elevated risk of halting effective trials by mistake when small samples are used in the phase 2 stage. Specifically, for the BPP approach, the probability of erroneously stopping early is 9.15% on average across scenarios when *n*_1_ = 200. This rate escalates to 19.79% when *n*_1_ = 50. Futhermore, given the precise and yet unknown dose–response is pivotal for dose selection [39], a relatively larger sample size is mandated at early stage when response rates across dosage groups are closely aligned. If the response rates are more distinguishable, fewer subjects might suffice.

This study presents certain limitations. Firstly, trials with CPEs strictly control type 1 errors, making it challenging to achieve a 2.5% type 1 error during futility stop boundary calibration. Consequently, we set the type 2 error rate below 0.2 for a specific scenario of *n*_1_ = 100, $\rho$ = 0 and *p*_*S*_ = *p*_*C*_ to derive a reliable boundary. Besides, this article focuses on the fundamental situation where four binary endpoints serve as CPEs. Yet, other contexts could involve continuous endpoints, time-to-event endpoints or vaccine efficacy (1-RR) as parameters of interest. Previous studies, such as those by Choi [17], Schmidli [25] and Kimani et al. [26] have explored BPP in single endpoint settings. Adapting their methods to trials with CPEs will be a task for future work. Additionally, while we did not include an early stop for efficacy rule in our study, supplementary Table 5–8 (see “Supplementary table”) offer probabilities for such early termination for both the BPP and CP approach, using efficacy boundaries of > 0.8 and > 0.9, respectively, offering a reference for potential early stops in future studies when an efficacy stop rule is incorporated.

In conclusion, our study deepens the comprehension of BPP in seamless phase 2/3 trials with multiple CPEs, shedding light on more streamlined clinical trial designs. Upcoming studies should delve into these methos across diverse endpoints and assess their real-world application, aiming to enhance the drug and vaccine development process in tandem with CPEs.

## Conclusions

In conclusion, this study highlights the advantages of the BPP approach in seamless phase 2/3 trials with multiple CPEs. We found out that for smaller phase 2 sample sizes, the BPP approach either matches or surpasses the CP approach in overall power when the experimental group is non-inferior to the control. Notably, in scenarios where *n*_1_ = 50, the overall power advantage of BPP over CP was as high as 8.54% when *ρ* = 0, providing a robust alternative in specific experimental setups. Yet, if the experimental group underperforms, the BPP demands larger sample sizes due to a reduced early stop probability, a difference that can be as substantial as 126.25 subjects when *ρ* = 0.6 and *n*_1_ = 50. Conversely, with larger phase 2 samples, the BPP method consistently shows a higher probability of accurately halting futile trials over the CP method, while still maintaining a competitive overall power. The early stoppage probability for BPP slightly exceeds that of CP, reaching a peak difference of 5.76% when *ρ* = 0 and *n*_1_ = 200, signifying a more resource-efficient approach in such scenarios. Moreover, our simulations demonstrated that both BPP and CP maintain type 1 error rates under 2.5%. This research augments the comprehension of BPP in seamless phase 2/3 trials with multiple CPEs. Going forward, future work should focus on extending these methods to trials with various types of endpoints, such as continuous, time-to-event, and vaccine efficacy (1-RR).

### Supplementary Information

**Additional file 1.** 

**Additional file 2:** **Supplementary Table 1.** The 16 outcomes concerning 4 co-primary endpoints. **Supplementary Table 2.** The effect of informative prior on BPP when the null hypothesis is true. **Supplementary Table 3.** The effect of informative prior on BPP when the null hypothesis is false. **Supplementary Table 4. **The performance of BPP and CP with different stop boundaries when  = 0 and *n*_1_=100. **Supplementary Table 5.** The performance of BPP in comparison with CP approach for common correlation coefficient among the 4 endpoints is  = (0, 0.3, 0.6) and sample size used for each group at phase 2 stage is *n*_1_ = 50 in the nine scenarios when the null hypothesis is false. **Supplementary Table 6.** The performance of BPP in comparison with CP approach for common correlation coefficient among the 4 endpoints is  = (0, 0.3, 0.6) and sample size used for each group at phase 2 stage is *n*_1_= 100 in the nine scenarios when the null hypothesis is false. **Supplementary Table 7.** The performance of BPP in comparison with CP approach for common correlation coefficient among the 4 endpoints is  = (0, 0.3, 0.6) and sample size used for each group at phase 2 stage is *n*_1_= 150 in the nine scenarios when the null hypothesis is false. **Supplementary Table 8.** The performance of BPP in comparison with CP approach for common correlation coefficient among the 4 endpoints is  = (0, 0.3, 0.6) and sample size used for each group at phase 2 stage is *n*_1_= 200 in the nine scenarios when the null hypothesis is false. **Supplementary Table 9. **Correct dose selection rates for phase 2 stage when null hypothesis is false. **Supplementary Table 10. **Correct dose selection rates for phase 2 stage when the null hypothesis is true.

**Additional file 3:** **Figure S1.** Histogram of trial success probabilities obtained by BPP or CP approach.

## Data Availability

The example data sets generated and analyzed during this research are available from the corresponding author on reasonable request.

## Abbreviations

CPEs: Co-primary endpoints
BPP: Bayesian predictive power
CP: Conditional power
JT: Jennison and turnbull
