# Supplementary material for: Seamless phase 2/3 design for trials with multiple co-primary endpoints using Bayesian predictive power
Source: BMC Med Res Methodol. 2024 Jan 17;24:12. doi: 10.1186/s12874-024-02144-2 (PMC10792895; doi:10.1186/s12874-024-02144-2)
Supplement: Supplementary file 1 — Additional file 1. [file 12874_2024_2144_MOESM1_ESM.docx]

1. ***Dirichlet-Multinomial model for trials with 4 Co-primary endpoints***

Suppose there is a quadrivalent meningococcal tetanus toxoid-conjugate vaccine trial with *n* subjects in each group. Four binary endpoints are used as co-primary endpoints (CPEs). A permutation based on the four endpoints produces 16 mutually exclusive outcomes that each subject may experience. The 16 outcomes are listed in supplementary table 1.

Let *Y_ik_* denote the response of subject *i* (*i* = 1, …, *n*) in terms of endpoint *k* (*k* = 1,2,3,4), *Y_ki_* = 1 represents positive response and *Y_ki_* = 0 represents negative response for endpoint *k*, subject *i*. Let *x_j_* denote the number of subjects who have experienced outcome *j* (*j*=1,..16)**,** such that $\sum_{j=1}^{16} x_{j}=n$. Let *p_j_* denote the probability of outcome *j*, such that $\sum_{j=1}^{16} p_{j}=1$.

The vectors of the probabilities corresponding to 16 outcomes are denoted by ***p*** = (*p*_1_, …, *p*_16_). The number of subjects who have experienced these outcomes are denoted by ***x*** = (*x*_1_, . . . ,*x*_16_). Thus, ***x*** follows a multinomial distribution with parameters *n* and ***p***. The probability mass function of this multinomial distribution is:

$$\begin{matrix} f\left( x_{1},\ldots,x_{16};n,p_{1},\ldots,p_{16} \right) & =\text{Pr}\left( X_{1}=x_{1}\text{ and }\ldots\text{ and }X_{16}=x_{16} \right) \\ & =\left\{ \begin{matrix} \frac{n!}{x_{1}!\cdots x_{16}!}p_{1}^{x_{1}}\times\cdots\times p_{16}^{x_{16}}, & \text{ when }\sum_{j=1}^{16} x_{j}=n \\ 0 & \text{ otherwise } \end{matrix} \right. \end{matrix}$$

As the conjugate prior of multinomial distribution, Dirichlet distribution is taken,

***p*** ∼ Dirichlet(*α*_1_, . . . , *α*_16_),

where ***a*** = (*a*_1_,…, *a*_16_) is the vextor of hyperparameters. Then the posterior distribution of ***p*** is parameterized by an updated vector ***a*’**,

***p*** | ***x*** ∼ Dirichlet(*α*_1_ + *x*_1_, . . . , *α_16_*+ *x_16_*),

where ***a***’ = (*α*_1_ + *x*_1_, . . . , *α_16_*+ *x_16_*). Here, *a*_1_ = … = *a*_16_ = 1 is taken with so that the same density to each value of the probability vector ***p*** is given. Thus, the posterior distribution of ***p*** is,

***p*** | ***x*** ∼ Dirichlet(1 + *x*_1_, . . . , 1+ *x_16_*),

Based on ***p*** | ***x*** , the marginal probabilities of seroresponse rates for group S is, ***p_S_*** = (*p_s_*_1_, …, *p_s_*_4_) for endpoint 1, 2, 3 and 4 can be calculated as,

*p_S_*_1_ = *p*_1_ + *p*_2_ + *p*_3_ + *p*_4_ + *p*_5_ + *p*_6_ + *p*_7_ + *p*_8_,

*p_S_*_2_ = *p*_1_ + *p*_2_ + *p*_3_ + *p*_4_ + *p*_9_ + *p*_10_ + *p*_11_ + *p*_12_,

*p_S_*_3_ = *p*_1_ + *p*_2_ + *p*_5_ + *p*_6_ + *p*_9_ + *p*_10_ + *p*_13_ + p_14_,

*p_S_*_4_ = *p*_1_ + *p*_3_ + *p*_5_ + *p*_7_ + *p*_9_ + *p*_11_ + *p*_13_ + *p*_15 ._

1. ***Possible choices of informative priors and their impact on the results***

In an attempt to better understand the influence of different prior selections on the operating characteristics of bayesian predictive power (BPP), we conducted a series of simulations. In the context of initiating a phase 2 trial, the investigational drug or biological product often lacks substantial prior information regarding its efficacy across different doses. Consequently, for the experimental groups, we adopt non-informative priors to reflect this uncertainty. However, for the control group, which typically has an established research foundation, informative priors are employed to incorporate existing knowledge. In our design, we assess the impact of prior information on outcomes by introducing two different scenarios for the control group: First, where the prior for the control group supports the non-inferiority hypothesis, ***p****_T_* - ***p****_C_* > -0.1, and second, where the prior does not back the non-inferiority hypothesis.

Suppose there is a clinical trial with four CPEs. Let the prior information of response rates, which comes from the historical control group, be denoted by ***p****_hc_* = (*p_hc_*_1_, *p_hc_*_2_, *p_hc_*_3_, *p_hc_*_4_). These probabilities correspond to the 16 outcomes in the Dirichlet-Multinomial model, as shown in Supplementary Table 1, where outcome 1 represents the situation where all endpoints have positive outcomes, outcomes 2, 3, 5, and 9 correspond to cases with three positive endpoints, outcomes 4, 6, 7, 10, 11, and 13 correspond to cases with two positive endpoints, outcomes 8, 12, 14, and 15 correspond to cases with only one positive endpoint, and outcome 16 represents the situation where all endpoints have negative outcomes. To estimate the hyperparameter vector ***α*** = (*α*_1_, ..., *α*_16_), the historical probabilities for each outcome, denoted as ***p*** = (*p*_1_, ..., *p*_16_), were needed. Let the sample size of the historical control group be *n_h_*; then, *n_h_*⋅*p* can be used as the initial estimate for the hyperparameter vector ***α***, where ***α*** = (*n_h_*⋅*p*_1_, ..., *n_h_*⋅*p*_16_).

Here, we generate simulated historical data for two distinct scenarios. In the scenario where the historical data support the non-inferiority hypothesis, we set ***p****_hc_* = ***p***_c_ - 0.1 = (0.3246, 0.3965, 0.3478, 0.3339). Based on the given ***p****_hc_*, we have:

*p_hc_*_1_ = *p*_1_ + *p*_2_ + *p*_3_ + *p*_4_ + *p*_5_ + *p*_6_ + *p*_7_ + *p*_8_,

*p_hc_*_2_ = *p*_1_ + *p*_2_ + *p*_3_ + *p*_4_ + *p*_9_ + *p*_10_ + *p*_11_ + *p*_12_,

*p_hc_*_3_ = *p*_1_ + *p*_2_ + *p*_5_ + *p*_6_ + *p*_9_ + *p*_10_ + *p*_13_ + *p*_14_,

*p_hc_*_4_ = *p*_1_ + *p*_3_ + *p*_5_ + *p*_7_ + *p*_9_ + *p*_11_ + *p*_13_ + *p*_15_.

Additionally, *p*_1_ + ... + *p*_16_ = 1.

Based on the five equations mentioned above, there are many possible combinations for the 16 unknown values in ***p***. One possible combination for ***p****_SUP_* is (0.23, 0.08, 0.01, 0, 0, 0, 0, 0, 0, 0, 0.06, 0, 0, 0.03, 0.03, 0.56). Similarly, to create a prior which does not support the non-inferior hypothesis, we can set the prior as ***p****_hc_* = *p_c_* + 0.1 = (0.5246, 0.5965, 0.5478, 0.5339). In this case, ***p****_NONSUP_* is (0, 0.13, 0.17, 0.07, 0.02, 0.06, 0.08, 0, 0.08, 0.07, 0.07, 0.01, 0.13, 0.05, 0, 0.06).

To evaluate the impact of different choices of prior on type 1 error for the BPP approach, we set ***p****_S_* - ***p***_c_ = -0.1, representing the situation where the null hypothesis is true. To assess the impact on power, we set ***p****_S_* = ***p****_c_*, representing the situation where the null hypothesis is false. The simulation results based on 10,000 iterations can be found in Supplementary Tables 2 and 3.

From Supplementary Table 2 and 3, we found that in clinical trials with CPEs, incorporating a prior that supports the non-inferiority hypothesis does not necessarily enhance the trial's power. In particular, when the sample size of historical control group is equal to that of current control group, such a prior may even reduce the study's power and increase the sample size by diminishing the chance of stopping an ineffective trial. This result occurs because, in the presence of CPEs, data that ostensibly supports the non-inferiority hypothesis may not genuinely do so. Taking *n_h_* =200 as an example, although the prior supporting the non-inferiority hypothesis, ***α*** = (*n_h_*⋅*p*_1_, ..., *n_h_*⋅*p*_16_) = (46, 16, 2, 0, 0, 0, 0, 0, 0, 0, 12, 0, 0, 6, 6, 112), is derived from relatively low response rates across all endpoints, the scenario in which all four endpoints simultaneously meet the criteria holds a relatively larger weight with *α*_1_ = *n_h_*⋅*p*_1_ = 46. Applying this historical control information as a "supportive" prior might weaken the study's power when compared to using a non-informative prior.

In conclusion, due to potential intricate interactions among multiple endpoints, if the objective of a study is to meet the requirements for all endpoints at the same time, incorporating prior information on multiple endpoints may inadvertently impact our evaluation of the final outcomes. Therefore, we recommend that readers carefully consider whether to use an informative prior when historical data is available. In the absence of historical data, utilizing a non-informative prior can be an appropriate approach.

1. ***Adjusted p-value calculation using the Holm’s sequentially rejective procedure at interim analysis in a seamless phase II/III trial with multiple dose groups***

For a seamless Phase II/III trial with three dose groups and a control group, the Holm’s sequentially rejective procedure^1^ can be applied to obtain the adjusted *p*-values for multiple comparisons. Note that any adjusted value greater than 1 should be set to 1.

To demonstrate non-inferiority of the trial at a one-sided significance level of *α* with a power of 1-*β*, the null hypothesis, *H*_(_*_i_*_)0_: $\pi_{i}-\pi_{C}= \delta$, is tested against the alternatives *H*_(i)1_: $\pi_{i}-\pi_{C}> \delta$, where *δ* is the non-inferiority margin, *i* denotes the dose group (1 = 1, …, *I*).

Suppose the original *p*-values obtained from interim analysis are *p*_1_, *p*_2_, …, *p_I_* corresponding to hypothesis *H*_(1)0_, *H*_(2)0_,…, *H*_(_*_I_*_)0_ with the order *p*_1_ ≤ *p*_2_ ≤…≤ *p_I_*. Using Holm’s sequentially rejective method with *α*, the following algorithm are taken:

1. If *p*_1_ > *α*/*I*, then accept all hypothesis *H*_(1)0_, *H*_(2)0_, …, *H*_(_*_I_*_)0_ and stop; otherwise, reject *H*_(1)0_ and continue.
2. If *p*_2_ > *α*/(*I* -1), then accept all hypothesis *H*_(2)0_, …, *H*_(_*_I_*_)0_ and stop; otherwise, reject *H*_(2)0_ and continue.

……

1. If *p_I_* > *α*, then accept hypothesis *H*_(_*_I_*_)0_; otherwise, reject *H*_(_*_I_*_)0_.

The corresponding adjusted *p*-values are computed as follows:

1. *p_adj_*_1_ = *I***p*_1_
2. *p_adj_*_2_ = max(*p_adj_*_1_, (*I* - 1)**p*_2_)

……

1. *p_adjI_* = max(*p_adj_*_(_*_I_*_-1)_, 1**p_I_*)

The adjusted *p*-values can be compared directly to the desired familywise significance level.

1. ***Combining p-values in seamless phase II/III trials using the inverse normal weighted combination test***

In a non-inferiority seamless phase II/III trial, the task is often to make an overall decision by integrating evidence from both phases. The inverse normal weighted combination test^2^ is a well-established method for combining p-values from different stages of such trials. The core idea behind the inverse normal weighted combination test is to first transform the *p*-values from each stage into their corresponding z-scores. Subsequently, these z-scores are weighted and combined to yield a composite z-score. This composite z-score can then be converted back into a combined *p*-value for decision-making purposes.

1. *Z*-score computation:

For a given *p*-value, the corresponding *z*-score is the quantile of the standard normal distribution.

$$Z_{j}=\Phi^{-1}\left( 1-p_{j} \right)$$

where $\Phi^{-1}$ denotes the quantile function of the standard normal distribution, and $p_{j}$ is the *p*-value from the *j*-th stage.

2. Weight calculation:

For multi-stage design, the weights can be computed using:

$$w_{j}=\sqrt{\frac{n_{j}}{n}}$$

Here, *n_j_* represents the sample size in the *j*-th stage, and *n* is the overall sample size.

3. *Z*-score combination:

For trials with 2 stages, the combined *z*-score can be obtained through:

$$Z_{\text{combined }}=\frac{w_{1}\times Z_{1}+w_{2}\times Z_{2}}{\sqrt{w_{1}^{2}+w_{2}^{2}}}$$

Given $w_{1}=\sqrt{\frac{n_{1}}{n}}$, $w_{2}=\sqrt{\frac{n_{2}}{n}}$ and *n* = *n*_1_ + *n*_2_, we have $\sqrt{w_{1}^{2}+w_{2}^{2}}$ = 1. Thus, $Z_{\text{combined }}$= $w_{1}\times Z_{1}+w_{2}\times Z_{2}$.

4. Combined *p*-value computation:

Given the one-sided nature of the test, the combined p-value is:

$$p_{\text{combined }}=1-\Phi\left( Z_{\text{combined }} \right)$$

Here, $\Phi$( ) represents the cumulative distribution function of the standard normal distribution.

**Reference**

1. Westfall P H, Young S S. Resampling-based multiple testing: Examples and methods for p-value adjustment[M]. John Wiley & Sons, 1993. pp. 62-75.
2. Lehmacher W, Wassmer G. Adaptive sample size calculations in group sequential trials[J]. Biometrics, 1999, 55(4): 1286-1290.
