# Supplementary material for: Seamless phase 2/3 design for trials with multiple co-primary endpoints using Bayesian predictive power
Source: BMC Med Res Methodol. 2024 Jan 17;24:12. doi: 10.1186/s12874-024-02144-2 (PMC10792895; doi:10.1186/s12874-024-02144-2)
Supplement: Supplementary file 2 — Additional file 2: Supplementary Table 1. The 16 outcomes concerning 4 co-primary endpoints. Supplementary Table 2. The effect of informative prior on BPP when the null hypothesis is true. Supplementary Table 3. The effect of informative prior on BPP when the null hypothesis is false. Supplementary Table 4. The performance of BPP and CP with different stop boundaries when = 0 and n1=100. Supplementary Table 5. The performance of BPP in comparison with CP approach for common correlation coefficient among the 4 endpoints is = (0, 0.3, 0.6) and sample size used for each group at phase 2 stage is n1 = 50 in the nine scenarios when the null hypothesis is false. Supplementary Table 6. The performance of BPP in comparison with CP approach for common correlation coefficient among the 4 endpoints is = (0, 0.3, 0.6) and sample size used for each group at phase 2 stage is n1= 100 in the nine scenarios when the null hypothesis is false. Supplementary Table 7. The performance of BPP in comparison with CP approach for common correlation coefficient among the 4 endpoints is = (0, 0.3, 0.6) and sample size used for each group at phase 2 stage is n1= 150 in the nine scenarios when the null hypothesis is false. Supplementary Table 8. The performance of BPP in comparison with CP approach for common correlation coefficient among the 4 endpoints is = (0, 0.3, 0.6) and sample size used for each group at phase 2 stage is n1= 200 in the nine scenarios when the null hypothesis is false. Supplementary Table 9. Correct dose selection rates for phase 2 stage when null hypothesis is false. Supplementary Table 10. Correct dose selection rates for phase 2 stage when the null hypothesis is true. [file 12874_2024_2144_MOESM2_ESM.docx]

**Supplementary Table 1** The 16 outcomes concerning 4 co-primary endpoints

| **Outcome** | **Endpoint 1** | **Endpoint 2** | **Endpoint 3** | **Endpoint 4** | ***x_j_*** | ***p_j_*** |
| --- | --- | --- | --- | --- | --- | --- |
| 1 | 1 | 1 | 1 | 1 | *x*_1_ | *p*_1_ |
| 2 | 1 | 1 | 1 | 0 | *x*_2_ | *p*_2_ |
| 3 | 1 | 1 | 0 | 1 | *x*_3_ | *p*_3_ |
| 4 | 1 | 1 | 0 | 0 | *x*_4_ | *p*_4_ |
| 5 | 1 | 0 | 1 | 1 | *x*_5_ | *p*_5_ |
| 6 | 1 | 0 | 1 | 0 | *x*_6_ | *p*_6_ |
| 7 | 1 | 0 | 0 | 1 | *x*_7_ | *p*_7_ |
| 8 | 1 | 0 | 0 | 0 | *x*_8_ | *p*_8_ |
| 9 | 0 | 1 | 1 | 1 | *x*_9_ | *p*_9_ |
| 10 | 0 | 1 | 1 | 0 | *x*_10_ | *p*_10_ |
| 11 | 0 | 1 | 0 | 1 | *x*_11_ | *p*_11_ |
| 12 | 0 | 1 | 0 | 0 | *x*_12_ | *p*_12_ |
| 13 | 0 | 0 | 1 | 1 | *x*_13_ | *p*_13_ |
| 14 | 0 | 0 | 1 | 0 | *x*_14_ | *p*_14_ |
| 15 | 0 | 0 | 0 | 1 | *x*_15_ | *p*_15_ |
| 16 | 0 | 0 | 0 | 0 | *x*_16_ | *p*_16_ |

**Supplementary Table 2** The effect of informative prior on BPP when the null hypothesis is true

| ***n_h_*** | **Type 1 error (%)** | | | **Stop Percentage(%)** | | | **Sample size** | | |
| --- | --- | --- | --- | --- | --- | --- | --- | --- | --- |
|  | **Sup** | **Non-Info*** | **Nonsup** | **Sup** | **Non-Info*** | **Nonsup** | **Sup** | **Non-Info*** | **Nonsup** |
| 5 | <0.01 | <0.01 | <0.01 | 94.53 | 93.89 | 92.24 | 282.05 | 291.65 | 316.40 |
| 10 | <0.01 | <0.01 | <0.01 | 96.16 | 93.89 | 92.05 | 257.60 | 291.65 | 319.25 |
| 20 | <0.01 | <0.01 | <0.01 | 98.23 | 93.89 | 92.20 | 226.55 | 291.65 | 317.00 |
| 50 | <0.01 | <0.01 | <0.01 | 99.85 | 93.89 | 91.62 | 202.25 | 291.65 | 325.70 |
| 100 | <0.01 | <0.01 | <0.01 | 100.00 | 93.89 | 91.36 | 200.00 | 291.65 | 329.60 |
| 200 | <0.01 | <0.01 | <0.01 | 100.00 | 93.89 | 92.08 | 200.00 | 291.65 | 318.80 |

Note: In each simulation, *n*_1_ = 200, *n*_2_ = 250, *β* = 0.20, *n*_min_ = 300, *n*_max_ = 1500, $\rho$ = 0, *α* = 0.025; 'Sup' represents the prior supporting the non-inferiority hypothesis; 'Nonsup' the prior not supporting the non-inferiority hypothesis; 'Non-Info' represents the non-informative prior; 'Non-Info' represents the non-informative prior, where the hyperparameters are assigned as *α*_1_ = ... = *α*_16_ = 1.

**Supplementary Table 3** The effect of informative prior on BPP when the null hypothesis is false

| ***n_h_*** | **Power (%)** | | | **Stop Percentage(%)** | | | **Sample size** | | |
| --- | --- | --- | --- | --- | --- | --- | --- | --- | --- |
|  | **Sup** | **Non-Info*** | **Nonsup** | **Sup** | **Non-Info*** | **Nonsup** | **Sup** | **Non-Info*** | **Nonsup** |
| 5 | 88.89 | 90.29 | 90.65 | 10.29 | 8.70 | 8.04 | 1484.50 | 1500.66 | 1494.08 |
| 10 | 86.88 | 90.29 | 91.34 | 12.27 | 8.70 | 7.54 | 1472.25 | 1500.66 | 1498.97 |
| 20 | 82.22 | 90.29 | 92.13 | 17.09 | 8.70 | 6.76 | 1422.50 | 1500.66 | 1506.72 |
| 50 | 63.02 | 90.29 | 93.96 | 36.05 | 8.70 | 4.89 | 1156.96 | 1500.66 | 1524.57 |
| 100 | 29.11 | 90.29 | 95.26 | 70.38 | 8.70 | 3.53 | 644.30 | 1500.66 | 1532.71 |
| 200 | 2.43 | 90.29 | 95.62 | 97.48 | 8.70 | 2.91 | 237.80 | 1500.66 | 1534.70 |

Note: In each simulation, *n*_1_ = 200, *n*_2_ = 250, *β* = 0.20, *n*_min_ = 300, *n*_max_ = 1500, $\rho$ = 0, *α* = 0.025; 'Sup' represents the prior supporting the non-inferiority hypothesis; 'Nonsup' the prior not supporting the non-inferiority hypothesis; 'Non-Info' represents the non-informative prior; 'Non-Info' represents the non-informative prior, where the hyperparameters are assigned as *α*_1_ = ... = *α*_16_ = 1.

**Supplementary Table 4** The performance of BPP and CP with different stop boundaries when $\rho$ = 0 and *n*_1_=100

| **Approach** | **Stop boundary *η*** | ***p****_S_* = ***p****_C_* | | ***p***_3_ - ***p****_C_* = -0.1 | |
| --- | --- | --- | --- | --- | --- |
|  |  | **Power(%)** | **Stop Percentage(%)** | **Type 1 Error (%)** | **Stop Percentage(%)** |
| BPP | 0.006 | 84.91 | 12.89 | <0.01 | 83.21 |
|  | 0.008 | 84.78 | 13.02 | <0.01 | 83.32 |
|  | **0.010** | **81.26** | **16.74** | **<0.01** | **87.81** |
|  | 0.015 | 80.54 | 17.48 | <0.01 | 88.57 |
|  | 0.020 | 77.60 | 20.67 | <0.01 | 90.37 |
| CP | 0.0016 | 83.22 | 12.91 | <0.01 | 83.67 |
|  | 0.0017 | 83.14 | 13.03 | <0.01 | 84.98 |
|  | **0.0018** | **81.21** | **15.12** | **<0.01** | **87.75** |
|  | 0.0019 | 78.97 | 17.48 | <0.01 | 88.57 |
|  | 0.0020 | 78.72 | 17.69 | <0.01 | 88.57 |

**Supplementary Table 5** The performance of BPP in comparison with CP approach for common correlation coefficient among the 4 endpoints is $\rho$ = (0, 0.3, 0.6) and sample size used for each group at phase 2 stage is *n*_1_ = 50 in the nine scenarios when the null hypothesis is false.

| **Scenario^*^** | **Diff^*^** | **Correlation** | **Power (%)** | | **Conditional Power(%)** | | | | | | **Stop Percentage(%)** | | ***n_total_*** | |
| --- | --- | --- | --- | --- | --- | --- | --- | --- | --- | --- | --- | --- | --- | --- |
|  |  |  |  |  | **Mean ±** **SD** | | **≥0.8** | | **≥0.9** | |  |  |  |  |
|  |  |  | **BPP** | **CP** | **BPP** | **CP** | **BPP** | **CP** | **BPP** | **CP** | **BPP** | **CP** | **BPP** | **CP** |
| *p_T_* < *p_C_* | (-0.02, -0.02, -0.02, -0.02) | 0 | 57.92 | 46.78 | 17.27±25.60 | 12.31±24.20 | 4.53 | 4.53 | 2.28 | 2.28 | 33.63 | 43.12 | 1045.06 | 894.40 |
|  |  | 0.3 | 62.34 | 52.39 | 26.09±32.49 | 21.01±32.37 | 11.38 | 11.38 | 7.25 | 7.33 | 27.64 | 34.97 | 1129.22 | 981.30 |
|  |  | 0.6 | 66.58 | 56.76 | 36.19±37.01 | 31.13±38.03 | 20.21 | 20.21 | 14.45 | 14.54 | 21.14 | 27.22 | 1206.64 | 1036.55 |
|  | (-0.02, -0.02, -0.02, 0) | 0 | 61.09 | 50.61 | 18.97±26.79 | 13.8±25.61 | 5.39 | 5.39 | 2.84 | 2.85 | 31.20 | 40.31 | 1081.48 | 934.31 |
|  |  | 0.3 | 64.64 | 55.33 | 27.84±33.21 | 22.64±33.26 | 12.35 | 12.35 | 8.02 | 8.09 | 25.82 | 32.69 | 1155.52 | 1009.09 |
|  |  | 0.6 | 67.79 | 58.70 | 37.94±37.39 | 32.83±38.60 | 21.72 | 21.71 | 15.71 | 15.77 | 19.75 | 25.56 | 1223.03 | 1051.56 |
|  | (-0.02, -0.02, 0, 0) | 0 | 64.39 | 54.31 | 20.72±27.83 | 15.32±26.85 | 6.32 | 6.31 | 3.31 | 3.33 | 28.49 | 37.51 | 1121.90 | 973.77 |
|  |  | 0.3 | 67.16 | 58.61 | 29.79±33.88 | 24.43±34.17 | 13.64 | 13.64 | 9.14 | 9.20 | 23.78 | 30.34 | 1184.31 | 1038.79 |
|  |  | 0.6 | 69.86 | 61.17 | 39.89±37.80 | 34.79±39.23 | 23.56 | 23.55 | 17.02 | 17.11 | 18.21 | 23.76 | 1242.21 | 1067.20 |
|  | (-0.02, 0, 0, 0) | 0 | 68.27 | 58.98 | 22.72±28.90 | 17.08±28.17 | 7.22 | 7.22 | 3.91 | 3.92 | 25.85 | 34.40 | 1161.15 | 1016.38 |
|  |  | 0.3 | 70.05 | 62.31 | 31.77±34.68 | 26.37±35.27 | 15.42 | 15.42 | 10.28 | 10.39 | 21.75 | 28.32 | 1213.14 | 1060.82 |
|  |  | 0.6 | 71.71 | 64.56 | 42.02±38.12 | 36.91±39.78 | 25.33 | 25.33 | 18.60 | 18.72 | 16.81 | 21.97 | 1259.18 | 1080.06 |
| *p_T_* = *p_C_* | (0, 0, 0, 0) | 0 | 71.83 | 63.36 | 24.92±29.97 | 19.07±29.52 | 8.41 | 8.40 | 4.70 | 4.72 | 23.14 | 31.65 | 1201.49 | 1052.86 |
|  |  | 0.3 | 72.75 | 66.09 | 34.04±35.34 | 28.52±36.19 | 16.92 | 16.92 | 11.80 | 11.86 | 19.51 | 25.80 | 1244.17 | 1089.08 |
|  |  | 0.6 | 74.26 | 68.24 | 44.37±38.38 | 39.30±40.26 | 27.48 | 27.48 | 20.27 | 20.34 | 14.94 | 19.97 | 1281.02 | 1095.61 |
| *p_T_* > *p_C_* | (0.02, 0, 0, 0) | 0 | 73.62 | 65.68 | 26.87±30.95 | 20.92±30.81 | 9.84 | 9.83 | 5.34 | 5.35 | 21.13 | 29.10 | 1231.22 | 1085.25 |
|  |  | 0.3 | 73.81 | 67.41 | 35.99±35.83 | 30.42±36.89 | 18.43 | 18.43 | 12.80 | 12.83 | 18.12 | 24.16 | 1261.60 | 1105.49 |
|  |  | 0.6 | 75.24 | 69.61 | 46.00±38.47 | 40.91±40.52 | 29.07 | 29.07 | 21.45 | 21.51 | 13.80 | 18.51 | 1293.43 | 1106.40 |
|  | (0.02, 0.02, 0, 0) | 0 | 75.40 | 68.37 | 29.03±31.78 | 22.92±31.90 | 11.13 | 11.11 | 6.19 | 6.20 | 19.24 | 26.28 | 1259.05 | 1122.51 |
|  |  | 0.3 | 75.29 | 69.05 | 38.14±36.38 | 32.58±37.71 | 20.40 | 20.39 | 14.24 | 14.29 | 16.41 | 22.45 | 1284.38 | 1121.07 |
|  |  | 0.6 | 76.59 | 71.13 | 47.99±38.60 | 42.97±40.84 | 30.91 | 30.91 | 23.19 | 23.26 | 12.65 | 16.92 | 1304.23 | 1113.69 |
|  | (0.02, 0.02, 0.02, 0) | 0 | 77.26 | 70.75 | 31.28±32.66 | 25.08±33.14 | 12.90 | 12.89 | 7.31 | 7.33 | 17.05 | 23.76 | 1291.25 | 1154.00 |
|  |  | 0.3 | 76.92 | 71.05 | 40.37±36.80 | 34.82±38.41 | 22.42 | 22.42 | 15.56 | 15.61 | 14.69 | 20.28 | 1306.01 | 1141.33 |
|  |  | 0.6 | 77.64 | 72.72 | 50.23±38.63 | 45.28±41.10 | 33.19 | 33.19 | 25.19 | 25.27 | 11.35 | 15.37 | 1316.41 | 1119.09 |
|  | (0.02, 0.02, 0.02, 0.02) | 0 | 78.71 | 72.83 | 33.73±33.43 | 27.44±34.28 | 14.80 | 14.80 | 8.59 | 8.63 | 15.05 | 21.27 | 1320.13 | 1183.50 |
|  |  | 0.3 | 78.14 | 72.98 | 42.99±37.20 | 37.46±39.09 | 24.92 | 24.92 | 17.45 | 17.51 | 13.11 | 18.43 | 1324.58 | 1155.25 |
|  |  | 0.6 | 78.87 | 74.85 | 52.74±38.56 | 47.88±41.29 | 36.03 | 36.03 | 27.36 | 27.42 | 10.01 | 13.70 | 1327.74 | 1125.71 |

Note: For scenario^*^, *T* represents expremential group; *C* represents control group**; *n_total_*** represents the sample size used for selected dose or control group during the whole trial.

**Supplementary Table 6** The performance of BPP in comparison with CP approach for common correlation coefficient among the 4 endpoints is $\rho$ = (0, 0.3, 0.6) and sample size used for each group at phase 2 stage is *n*_1_ = 100 in the nine scenarios when the null hypothesis is false.

| **Scenario^*^** | **Diff^*^** | **Correlation** | **Power (%)** | | **Conditional Power(%)** | | | | | | **Stop Percentage(%)** | | ***n_total_*** | |
| --- | --- | --- | --- | --- | --- | --- | --- | --- | --- | --- | --- | --- | --- | --- |
|  |  |  |  |  | **Mean ±** **SD** | | **≥0.8** | | **≥0.9** | |  |  |  |  |
|  |  |  | **BPP** | **CP** | **BPP** | **CP** | **BPP** | **CP** | **BPP** | **CP** | **BPP** | **CP** | **BPP** | **CP** |
| *p_T_* < *p_C_* | (-0.02, -0.02, -0.02, -0.02) | 0 | 64.66 | 59.39 | 19.84±25.65 | 16.18±24.84 | 5.28 | 4.97 | 1.99 | 2.00 | 28.25 | 26.51 | 1172.75 | 1182.38 |
|  |  | 0.3 | 68.00 | 62.35 | 27.66±31.37 | 24.04±31.34 | 11.56 | 11.20 | 6.34 | 6.39 | 24.39 | 22.88 | 1212.55 | 1189.43 |
|  |  | 0.6 | 71.83 | 66.26 | 36.94±35.73 | 33.51±36.44 | 20.84 | 20.19 | 13.11 | 13.20 | 19.16 | 18.02 | 1251.14 | 1187.70 |
|  | (-0.02, -0.02, -0.02, 0) | 0 | 68.40 | 63.90 | 22.07±26.95 | 18.28±26.34 | 6.50 | 6.08 | 2.58 | 2.59 | 25.64 | 23.80 | 1210.93 | 1216.88 |
|  |  | 0.3 | 70.83 | 66.11 | 29.97±32.36 | 26.30±32.53 | 13.28 | 12.80 | 7.52 | 7.56 | 22.02 | 20.38 | 1243.62 | 1214.60 |
|  |  | 0.6 | 73.67 | 69.13 | 39.08±36.22 | 35.67±37.08 | 22.84 | 22.13 | 14.44 | 14.49 | 17.34 | 16.19 | 1268.99 | 1200.68 |
|  | (-0.02, -0.02, 0, 0) | 0 | 72.01 | 69.01 | 24.59±28.33 | 20.68±27.96 | 8.16 | 7.59 | 3.25 | 3.31 | 22.86 | 20.98 | 1250.78 | 1251.90 |
|  |  | 0.3 | 73.69 | 70.44 | 32.61±33.23 | 28.90±33.59 | 15.27 | 14.72 | 8.66 | 8.76 | 19.80 | 18.22 | 1270.41 | 1234.31 |
|  |  | 0.6 | 76.65 | 72.24 | 41.72±36.68 | 38.30±37.72 | 25.30 | 24.37 | 16.54 | 16.65 | 15.36 | 14.32 | 1287.28 | 1206.68 |
|  | (-0.02, 0, 0, 0) | 0 | 76.96 | 75.43 | 27.52±29.46 | 23.45±29.34 | 9.76 | 9.21 | 4.23 | 4.25 | 19.59 | 18.11 | 1297.78 | 1285.34 |
|  |  | 0.3 | 78.01 | 75.24 | 35.58±34.04 | 31.83±34.63 | 17.79 | 17.13 | 10.13 | 10.23 | 16.90 | 15.88 | 1306.61 | 1253.49 |
|  |  | 0.6 | 79.80 | 76.55 | 44.83±37.03 | 41.46±38.28 | 28.27 | 27.35 | 18.75 | 18.91 | 13.22 | 12.43 | 1305.40 | 1209.54 |
| *p_T_* = *p_C_* | (0, 0, 0, 0) | 0 | 81.18 | 81.20 | 30.81±30.78 | 26.67±30.97 | 12.20 | 11.43 | 5.39 | 5.48 | 16.77 | 15.04 | 1337.09 | 1318.29 |
|  |  | 0.3 | 81.53 | 80.67 | 38.93±34.85 | 35.21±35.72 | 20.79 | 19.94 | 12.05 | 12.17 | 14.52 | 13.26 | 1331.70 | 1271.41 |
|  |  | 0.6 | 82.83 | 81.45 | 48.14±37.16 | 44.83±38.62 | 31.62 | 30.46 | 21.18 | 21.28 | 11.52 | 10.42 | 1313.13 | 1211.82 |
| *p_T_* > *p_C_* | (0.02, 0, 0, 0) | 0 | 83.09 | 83.46 | 33.40±31.68 | 29.23±32.12 | 14.35 | 13.43 | 6.59 | 6.61 | 14.84 | 13.01 | 1362.13 | 1336.10 |
|  |  | 0.3 | 82.92 | 82.44 | 41.37±35.34 | 37.67±36.41 | 23.17 | 22.08 | 14.04 | 14.13 | 13.07 | 11.54 | 1344.15 | 1276.47 |
|  |  | 0.6 | 83.57 | 82.48 | 50.15±37.22 | 46.88±38.80 | 33.82 | 32.43 | 23.02 | 23.03 | 10.29 | 9.23 | 1318.87 | 1208.93 |
|  | (0.02, 0.02, 0, 0) | 0 | 85.13 | 85.37 | 36.54±32.49 | 32.37±33.21 | 16.76 | 15.80 | 7.99 | 7.98 | 12.74 | 11.40 | 1388.39 | 1345.32 |
|  |  | 0.3 | 84.60 | 83.90 | 44.37±35.64 | 40.69±36.93 | 25.87 | 24.83 | 16.02 | 16.15 | 11.17 | 10.13 | 1361.65 | 1275.77 |
|  |  | 0.6 | 84.94 | 83.91 | 52.87±37.13 | 49.67±38.87 | 36.64 | 35.56 | 25.29 | 25.38 | 8.88 | 8.10 | 1323.13 | 1199.71 |
|  | (0.02, 0.02, 0.02, 0) | 0 | 86.92 | 87.38 | 39.91±33.27 | 35.76±34.28 | 19.68 | 18.55 | 9.93 | 9.93 | 10.65 | 9.39 | 1412.20 | 1354.78 |
|  |  | 0.3 | 86.28 | 85.85 | 47.60±35.87 | 44.00±37.38 | 29.14 | 27.79 | 18.34 | 18.51 | 9.40 | 8.42 | 1375.61 | 1275.57 |
|  |  | 0.6 | 86.50 | 85.86 | 55.93±36.92 | 52.85±38.81 | 40.22 | 38.76 | 28.28 | 28.36 | 7.59 | 6.82 | 1319.38 | 1184.80 |
|  | (0.02, 0.02, 0.02, 0.02) | 0 | 89.09 | 89.57 | 43.58±33.74 | 39.50±35.03 | 22.92 | 21.56 | 12.08 | 12.13 | 8.50 | 7.38 | 1435.11 | 1361.50 |
|  |  | 0.3 | 88.09 | 88.03 | 51.19±35.90 | 47.69±37.66 | 33.22 | 31.67 | 21.21 | 21.32 | 7.60 | 6.73 | 1386.08 | 1268.87 |
|  |  | 0.6 | 88.39 | 88.42 | 59.36±36.44 | 56.44±38.49 | 44.30 | 42.68 | 31.63 | 31.68 | 6.23 | 5.44 | 1313.64 | 1167.24 |

Note: For scenario^*^, *T* represents expremential group; *C* represents control group**; *n_total_*** represents the sample size used for selected dose or control group during the whole trial.

**Supplementary Table 7** The performance of BPP in comparison with CP approach for common correlation coefficient among the 4 endpoints is $\rho$ = (0, 0.3, 0.6) and sample size used for each group at phase 2 stage is *n*_1_ = 150 in the nine scenarios when the null hypothesis is false.

| **Scenario^*^** | **Diff^*^** | **Correlation** | **Power (%)** | | **Conditional Power(%)** | | | | | | **Stop Percentage(%)** | | ***n_total_*** | |
| --- | --- | --- | --- | --- | --- | --- | --- | --- | --- | --- | --- | --- | --- | --- |
|  |  |  |  |  | **Mean ±** **SD** | | **≥0.8** | | **≥0.9** | |  |  |  |  |
|  |  |  | **BPP** | **CP** | **BPP** | **CP** | **BPP** | **CP** | **BPP** | **CP** | **BPP** | **CP** | **BPP** | **CP** |
| *p_T_* < *p_C_* | (-0.02, -0.02, -0.02, -0.02) | 0 | 71.19 | 66.71 | 21.34±25.40 | 18.36±24.79 | 4.63 | 3.61 | 1.74 | 1.86 | 21.04 | 16.90 | 1328.09 | 1366.78 |
|  |  | 0.3 | 73.24 | 68.84 | 29.21±31.37 | 26.34±31.41 | 11.97 | 10.38 | 7.10 | 7.03 | 18.55 | 15.29 | 1332.66 | 1324.11 |
|  |  | 0.6 | 75.99 | 72.01 | 38.37±35.44 | 35.73±35.99 | 20.59 | 18.73 | 13.88 | 14.06 | 15.29 | 12.17 | 1321.80 | 1282.49 |
|  | (-0.02, -0.02, -0.02, 0) | 0 | 75.08 | 71.29 | 23.95±27.02 | 20.89±26.64 | 6.23 | 4.96 | 2.42 | 2.50 | 18.25 | 14.73 | 1366.05 | 1387.96 |
|  |  | 0.3 | 76.16 | 72.54 | 31.79±32.30 | 28.90±32.50 | 13.53 | 11.94 | 8.37 | 8.26 | 16.54 | 13.60 | 1352.22 | 1332.65 |
|  |  | 0.6 | 78.19 | 74.47 | 40.76±35.96 | 38.15±36.62 | 22.83 | 20.74 | 15.62 | 15.77 | 13.65 | 10.77 | 1331.75 | 1282.05 |
|  | (-0.02, -0.02, 0, 0) | 0 | 79.35 | 75.93 | 26.95±28.42 | 23.79±28.23 | 7.81 | 6.42 | 3.49 | 3.46 | 15.70 | 12.74 | 1399.08 | 1404.49 |
|  |  | 0.3 | 79.69 | 76.81 | 34.82±33.15 | 31.90±33.52 | 15.77 | 14.07 | 9.87 | 9.80 | 14.36 | 11.63 | 1372.83 | 1341.03 |
|  |  | 0.6 | 80.89 | 77.53 | 43.73±36.39 | 41.13±37.20 | 25.36 | 23.24 | 17.66 | 17.69 | 11.72 | 9.38 | 1339.80 | 1275.76 |
|  | (-0.02, 0, 0, 0) | 0 | 84.00 | 82.04 | 30.76±29.96 | 27.55±30.05 | 10.31 | 8.70 | 5.01 | 4.98 | 12.97 | 10.33 | 1433.23 | 1419.48 |
|  |  | 0.3 | 84.10 | 82.26 | 38.55±34.01 | 35.61±34.59 | 18.69 | 16.72 | 12.42 | 12.34 | 11.98 | 9.58 | 1390.71 | 1342.93 |
|  |  | 0.6 | 84.05 | 82.19 | 47.37±36.68 | 44.83±37.66 | 29.24 | 26.72 | 20.80 | 20.86 | 9.77 | 7.81 | 1345.28 | 1262.30 |
| *p_T_* = *p_C_* | (0, 0, 0, 0) | 0 | 88.22 | 88.18 | 34.99±31.09 | 31.70±31.46 | 13.16 | 11.40 | 7.01 | 6.83 | 10.42 | 8.13 | 1461.60 | 1426.65 |
|  |  | 0.3 | 87.71 | 87.63 | 42.75±34.66 | 39.85±35.49 | 22.34 | 20.00 | 15.11 | 15.10 | 9.58 | 7.30 | 1403.92 | 1341.81 |
|  |  | 0.6 | 88.00 | 87.29 | 51.45±36.70 | 48.98±37.87 | 32.89 | 30.43 | 24.52 | 24.53 | 7.74 | 6.16 | 1342.07 | 1242.27 |
| *p_T_* > *p_C_* | (0.02, 0, 0, 0) | 0 | 89.41 | 89.80 | 38.37±31.91 | 35.12±32.46 | 15.55 | 13.43 | 8.49 | 8.35 | 9.07 | 6.83 | 1470.21 | 1425.28 |
|  |  | 0.3 | 88.95 | 88.96 | 45.71±35.08 | 42.87±36.05 | 25.06 | 22.72 | 17.61 | 17.44 | 8.32 | 6.38 | 1404.99 | 1326.01 |
|  |  | 0.6 | 89.12 | 88.35 | 53.75±36.62 | 51.33±37.88 | 35.29 | 32.96 | 26.40 | 26.42 | 6.71 | 5.39 | 1335.49 | 1226.74 |
|  | (0.02, 0.02, 0, 0) | 0 | 91.27 | 91.34 | 42.13±32.63 | 38.93±33.42 | 19.25 | 16.69 | 10.88 | 10.85 | 7.27 | 5.49 | 1481.25 | 1413.57 |
|  |  | 0.3 | 90.47 | 90.56 | 49.23±35.26 | 46.48±36.40 | 28.93 | 26.07 | 20.06 | 20.06 | 6.99 | 5.24 | 1402.40 | 1309.60 |
|  |  | 0.6 | 90.09 | 89.64 | 56.84±36.39 | 54.53±37.78 | 38.95 | 36.49 | 29.99 | 30.16 | 5.84 | 4.49 | 1319.37 | 1200.78 |
|  | (0.02, 0.02, 0.02, 0) | 0 | 92.56 | 93.15 | 46.13±33.08 | 43.03±34.11 | 22.81 | 20.13 | 13.65 | 13.52 | 5.88 | 4.19 | 1482.04 | 1397.28 |
|  |  | 0.3 | 91.82 | 92.33 | 53.03±35.22 | 50.40±36.54 | 32.84 | 29.89 | 23.26 | 23.25 | 5.51 | 4.05 | 1397.00 | 1287.17 |
|  |  | 0.6 | 91.15 | 91.19 | 60.29±35.82 | 58.07±37.34 | 42.96 | 40.29 | 33.79 | 33.74 | 4.65 | 3.61 | 1300.04 | 1169.93 |
|  | (0.02, 0.02, 0.02, 0.02) | 0 | 94.19 | 95.00 | 50.64±33.23 | 47.65±34.50 | 27.54 | 24.25 | 17.39 | 17.20 | 4.57 | 3.07 | 1475.89 | 1369.17 |
|  |  | 0.3 | 93.47 | 94.03 | 57.36±34.85 | 54.89±36.32 | 37.59 | 34.29 | 27.44 | 27.17 | 4.23 | 3.03 | 1382.11 | 1252.60 |
|  |  | 0.6 | 93.03 | 93.41 | 64.44±34.92 | 62.38±36.57 | 48.07 | 45.06 | 38.28 | 38.12 | 3.60 | 2.72 | 1271.93 | 1127.82 |

Note: For scenario^*^, *T* represents expremential group; *C* represents control group**; *n_total_*** represents the sample size used for selected dose or control group during the whole trial.

**Supplementary Table 8** The performance of BPP in comparison with CP approach for common correlation coefficient among the 4 endpoints is $\rho$ = (0, 0.3, 0.6) and sample size used for each group at phase 2 stage is *n*_1_ = 200 in the nine scenarios when the null hypothesis is false.

| **Scenario^*^** | **Diff^*^** | **Correlation** | **Power (%)** | | **Conditional Power(%)** | | | | | | **Stop Percentage(%)** | | ***n_total_*** | |
| --- | --- | --- | --- | --- | --- | --- | --- | --- | --- | --- | --- | --- | --- | --- |
|  |  |  |  |  | **Mean ±** **SD** | | **≥0.8** | | **≥0.9** | |  |  |  |  |
|  |  |  | **BPP** | **CP** | **BPP** | **CP** | **BPP** | **CP** | **BPP** | **CP** | **BPP** | **CP** | **BPP** | **CP** |
| *p_T_* < *p_C_* | (-0.02, -0.02, -0.02, -0.02) | 0 | 72.34 | 68.79 | 22.37±25.65 | 19.80±25.15 | 4.80 | 4.02 | 1.93 | 1.88 | 20.23 | 14.47 | 1379.86 | 1434.84 |
|  |  | 0.3 | 75.20 | 70.95 | 30.40±31.55 | 27.97±31.57 | 12.32 | 10.87 | 6.64 | 6.48 | 17.40 | 12.79 | 1376.00 | 1381.04 |
|  |  | 0.6 | 78.18 | 74.42 | 39.03±35.23 | 36.73±35.68 | 20.75 | 19.31 | 13.33 | 13.09 | 14.23 | 10.45 | 1350.66 | 1319.84 |
|  | (-0.02, -0.02, -0.02, 0) | 0 | 76.56 | 73.42 | 25.37±27.19 | 22.72±26.86 | 6.36 | 5.39 | 2.70 | 2.61 | 17.61 | 12.45 | 1412.33 | 1448.76 |
|  |  | 0.3 | 77.80 | 74.52 | 33.26±32.44 | 30.80±32.59 | 14.13 | 12.50 | 7.92 | 7.83 | 15.49 | 11.34 | 1389.96 | 1381.78 |
|  |  | 0.6 | 80.08 | 77.03 | 41.64±35.73 | 39.38±36.29 | 23.21 | 21.45 | 15.09 | 14.86 | 12.71 | 9.36 | 1354.55 | 1310.20 |
|  | (-0.02, -0.02, 0, 0) | 0 | 81.04 | 78.55 | 29.17±28.90 | 26.46±28.79 | 8.67 | 7.43 | 3.89 | 3.79 | 14.64 | 10.36 | 1445.99 | 1456.64 |
|  |  | 0.3 | 81.50 | 79.21 | 36.87±33.43 | 34.39±33.78 | 17.08 | 15.42 | 10.08 | 9.94 | 13.33 | 9.45 | 1399.95 | 1378.09 |
|  |  | 0.6 | 82.65 | 80.15 | 44.98±36.14 | 42.75±36.84 | 26.36 | 24.58 | 17.53 | 17.37 | 10.96 | 7.86 | 1353.64 | 1297.49 |
|  | (-0.02, 0, 0, 0) | 0 | 85.60 | 84.36 | 33.57±30.38 | 30.82±30.51 | 11.57 | 10.01 | 5.35 | 5.24 | 11.56 | 8.05 | 1477.70 | 1461.64 |
|  |  | 0.3 | 85.31 | 84.17 | 41.16±34.27 | 38.71±34.82 | 20.78 | 18.93 | 12.84 | 12.64 | 11.01 | 7.57 | 1406.81 | 1365.72 |
|  |  | 0.6 | 86.32 | 84.51 | 49.19±36.44 | 47.02±37.30 | 30.70 | 28.82 | 21.03 | 20.84 | 8.67 | 6.21 | 1349.51 | 1273.61 |
| *p_T_* = *p_C_* | (0, 0, 0, 0) | 0 | 90.29 | 90.30 | 38.53±31.47 | 35.76±31.87 | 15.24 | 13.16 | 7.37 | 7.23 | 8.70 | 5.86 | 1500.66 | 1456.50 |
|  |  | 0.3 | 90.01 | 90.18 | 46.15±34.64 | 43.73±35.41 | 25.06 | 22.78 | 15.91 | 15.67 | 8.18 | 5.53 | 1415.90 | 1348.92 |
|  |  | 0.6 | 91.04 | 90.76 | 54.17±36.17 | 52.08±37.21 | 35.55 | 33.27 | 25.62 | 25.39 | 6.36 | 4.25 | 1335.81 | 1245.70 |
| *p_T_* > *p_C_* | (0.02, 0, 0, 0) | 0 | 91.60 | 91.76 | 42.03±32.16 | 39.31±32.73 | 18.22 | 15.92 | 9.27 | 9.17 | 7.43 | 4.89 | 1500.31 | 1438.75 |
|  |  | 0.3 | 91.27 | 91.44 | 49.29±34.82 | 46.92±35.71 | 28.17 | 25.63 | 18.41 | 18.26 | 6.99 | 4.55 | 1405.93 | 1328.40 |
|  |  | 0.6 | 91.97 | 91.71 | 56.74±36.00 | 54.73±37.12 | 38.53 | 36.06 | 28.13 | 27.97 | 5.36 | 3.64 | 1322.22 | 1220.62 |
|  | (0.02, 0.02, 0, 0) | 0 | 93.20 | 93.20 | 46.35±32.78 | 43.72±33.58 | 22.72 | 20.20 | 12.19 | 12.06 | 5.88 | 3.84 | 1493.95 | 1406.58 |
|  |  | 0.3 | 92.73 | 93.13 | 52.98±34.87 | 50.71±35.92 | 32.36 | 29.82 | 21.86 | 21.64 | 5.54 | 3.38 | 1391.10 | 1298.75 |
|  |  | 0.6 | 93.01 | 93.05 | 59.97±35.51 | 58.04±36.73 | 42.30 | 39.55 | 31.60 | 31.24 | 4.40 | 2.87 | 1298.03 | 1190.70 |
|  | (0.02, 0.02, 0.02, 0) | 0 | 94.63 | 94.99 | 51.11±32.99 | 48.61±33.99 | 27.73 | 24.89 | 15.94 | 15.76 | 4.36 | 2.82 | 1478.40 | 1366.14 |
|  |  | 0.3 | 94.33 | 94.69 | 57.36±34.51 | 55.21±35.71 | 37.04 | 34.50 | 26.01 | 25.73 | 4.12 | 2.57 | 1367.72 | 1254.92 |
|  |  | 0.6 | 94.38 | 94.52 | 64.06±34.69 | 62.27±36.01 | 47.15 | 44.36 | 36.11 | 35.88 | 3.45 | 2.20 | 1260.95 | 1144.06 |
|  | (0.02, 0.02, 0.02, 0.02) | 0 | 96.09 | 96.72 | 56.48±32.65 | 54.13±33.86 | 33.68 | 30.47 | 20.42 | 20.28 | 3.02 | 1.91 | 1451.95 | 1313.61 |
|  |  | 0.3 | 95.75 | 96.43 | 62.48±33.54 | 60.49±34.88 | 43.38 | 40.42 | 30.88 | 30.60 | 2.95 | 1.78 | 1333.37 | 1199.08 |
|  |  | 0.6 | 95.85 | 96.15 | 68.73±33.30 | 67.12±34.71 | 53.01 | 50.41 | 41.73 | 41.50 | 2.38 | 1.56 | 1213.39 | 1082.76 |

Note: For scenario^*^, *T* represents expremential group; *C* represents control group**;** ***n_total_*** represents the sample size used for selected dose or control group during the whole trial.

**Supplementary Table 9** Correct dose selection rates for phase 2 stage when null hypothesis is false

| **Scenario*** | | **Correlation** | **Correct dose selection (%)** | | | |
| --- | --- | --- | --- | --- | --- | --- |
|  |  |  | ***n*_1_ = 50** | ***n*­_1_ = 100** | ***n*­_1_ = 150** | ***n*­_1_ = 200** |
| *p_T_* < *p_C_* | (-0.02, -0.02, -0.02, -0.02) | 0 | 91.17 | 96.70 | 98.76 | 99.55 |
|  |  | 0.3 | 89.68 | 95.53 | 98.20 | 99.36 |
|  |  | 0.6 | 87.62 | 94.66 | 97.31 | 98.91 |
|  | (-0.02, -0.02, -0.02, 0) | 0 | 91.02 | 96.65 | 98.67 | 99.57 |
|  |  | 0.3 | 89.61 | 95.47 | 98.18 | 99.39 |
|  |  | 0.6 | 87.52 | 94.61 | 97.34 | 98.97 |
|  | (-0.02, -0.02, 0, 0) | 0 | 90.95 | 96.63 | 98.82 | 99.55 |
|  |  | 0.3 | 89.39 | 95.30 | 98.19 | 99.33 |
|  |  | 0.6 | 87.52 | 94.51 | 97.32 | 98.90 |
|  | (-0.02, 0, 0, 0) | 0 | 91.06 | 96.79 | 98.84 | 99.53 |
|  |  | 0.3 | 89.35 | 95.27 | 98.24 | 99.22 |
|  |  | 0.6 | 87.43 | 94.45 | 97.27 | 98.90 |
| *p_T_* = *p_C_* | (0, 0, 0, 0) | 0 | 90.86 | 96.70 | 98.78 | 99.56 |
|  |  | 0.3 | 89.07 | 95.42 | 98.00 | 99.24 |
|  |  | 0.6 | 87.48 | 94.45 | 97.09 | 98.93 |
| *p_T_* > *p_C_* | (0.02, 0, 0, 0) | 0 | 90.87 | 96.69 | 98.72 | 99.56 |
|  |  | 0.3 | 88.89 | 95.48 | 97.87 | 99.26 |
|  |  | 0.6 | 87.42 | 94.30 | 97.12 | 98.79 |
|  | (0.02, 0.02, 0, 0) | 0 | 91.03 | 96.78 | 98.62 | 99.46 |
|  |  | 0.3 | 88.86 | 95.35 | 97.89 | 99.22 |
|  |  | 0.6 | 87.52 | 94.17 | 97.04 | 98.77 |
|  | (0.02, 0.02, 0.02, 0) | 0 | 90.86 | 96.66 | 98.58 | 99.50 |
|  |  | 0.3 | 88.97 | 95.23 | 97.83 | 99.21 |
|  |  | 0.6 | 87.52 | 94.34 | 97.08 | 98.84 |
|  | (0.02, 0.02, 0.02, 0.02) | 0 | 90.64 | 96.68 | 98.60 | 99.56 |
|  |  | 0.3 | 88.88 | 95.27 | 97.84 | 99.14 |
|  |  | 0.6 | 87.31 | 94.33 | 97.19 | 98.77 |

Note: For scenario^*^, *T* represents expremential group; *C* represents control group**;**

**Supplementary Table 10** Correct dose selection rates for phase 2 stage when the null hypothesis is true

| **Correlation** | **Correct dose selection (%)** | | | |
| --- | --- | --- | --- | --- |
|  | ***n*_1_ = 50** | ***n*­_1_ = 100** | ***n*­_1_ = 150** | ***n*­_1_ = 200** |
| 0 | 91.91 | 97.35 | 99.08 | 99.67 |
| 0.3 | 90.57 | 96.30 | 98.65 | 99.39 |
| 0.6 | 89.30 | 95.05 | 97.89 | 99.23 |
