# Supplementary material for: Seamless phase 2/3 design for trials with multiple co-primary endpoints using Bayesian predictive power
Source: BMC Med Res Methodol. 2024 Jan 17;24:12. doi: 10.1186/s12874-024-02144-2 (PMC10792895; doi:10.1186/s12874-024-02144-2)
Supplement: Supplementary file 3 — Additional file 3: Figure S1. Histogram of trial success probabilities obtained by BPP or CP approach. [file 12874_2024_2144_MOESM3_ESM.docx]

**Figure S1** Histogram of trial success probabilities obtained by BPP or CP approach.


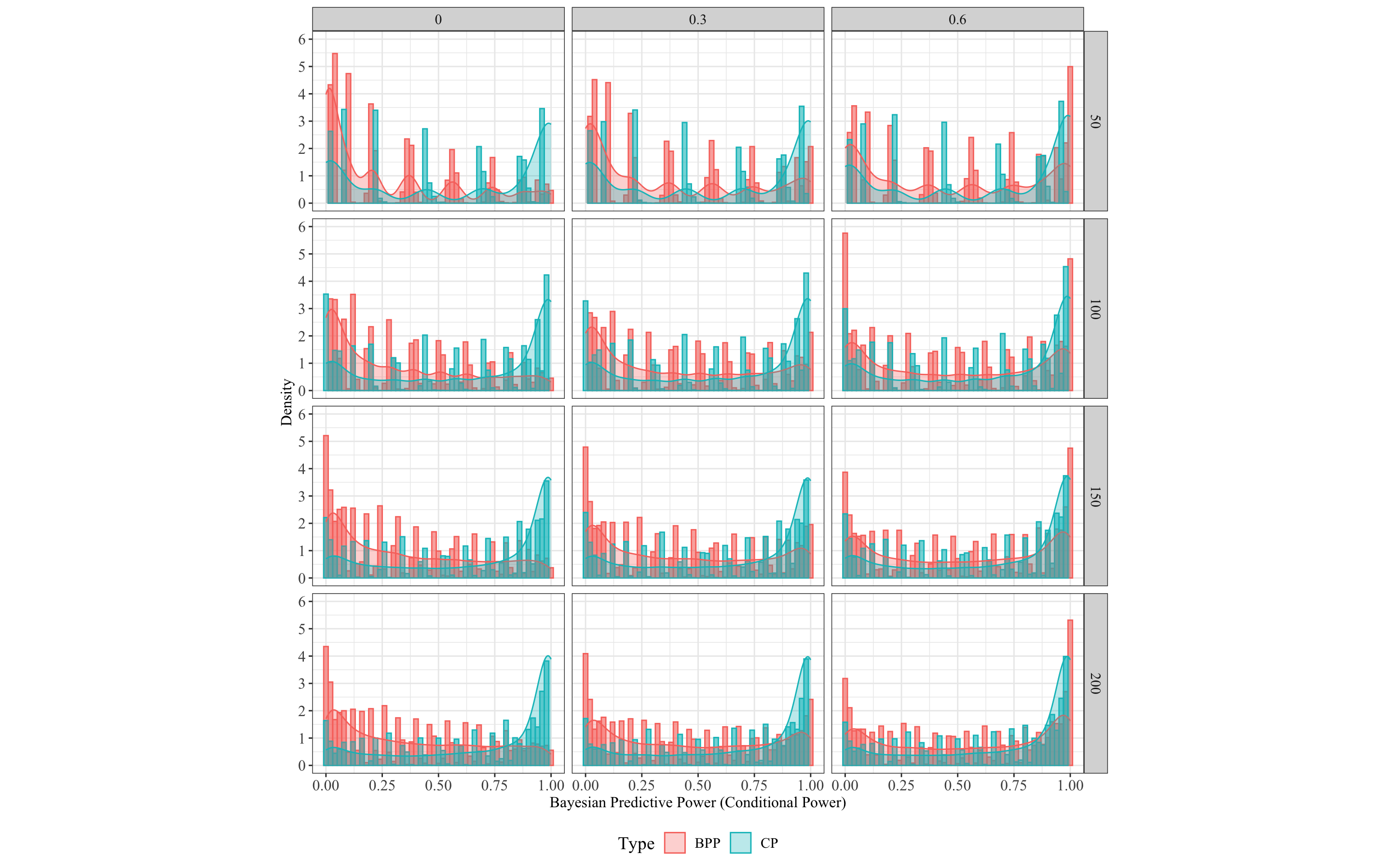


Note: The probabilities are calculated based on simulated data collected from phase 2 stage for common correlation coefficient among the 4 endpoints is $\rho$ = (0, 0.3, 0.6) (top legend) and sample size used for each group at phase 2 stage is *n*_1_ = (50, 100, 150, 200) (right legend) when the seroresponse difference between experimental vaccine and control vaccine is (0, 0, 0, 0).
